# Maximal independence and regularity of Al/Si distributions in disordered phyllosilicates 2:1

**DOI:** 10.1107/S2053273326009794

**Published:** 2026-10-15

**Authors:** Montauban Moreira de Oliveira Jr, Jean-Guillaume Eon

**Affiliations:** a Instituto de Ciências Exatas, Matemática Pura, Universidade Federal Rural do Rio de Janeiro, P1-90-4, Seropédica, Rio de Janeiro 23890-000, Brazil; b Instituto de Química, Universidade Federal do Rio de Janeiro, Avenida Athos da Silveira Ramos, 149 Bloco A, Cidade Universitária, Rio de Janeiro 21941-909, Brazil; Institute of Crystallography - CNR, Bari, Italy

**Keywords:** nets, quotient graphs, disorder, phyllosilicates

## Abstract

As an extension of previous works, we analyse regularity in disordered Al/Si distributions in phyllosilicates 2:1. The method is based on an analysis of maximal independent sets in a labelled quotient graph of the net. The results, similar to those obtained through the principle of homogeneous dispersion, are in quantitative agreement with published ^29^Si MAS NMR intensity data for muscovite, phlogopite and vermiculite. The method also allows an easy construction of structural models considering disorder and non-stoichiometry.

## Abstract 2

The distribution of aluminium cations in disordered phyllosilicates is analysed according to the concepts of maximal independence and regularity.

## 1. Introduction

Several papers have discussed structural models for Al/Si disorder in phyllosilicates 2:1 (see Herrero *et al.*, 1985 ▸; Herrero *et al.*, 1986 ▸; Herrero *et al.*, 1987 ▸; Herrero *et al.*, 1989 ▸; Herrero & Sanz, 1991 ▸, and references therein). These works show in particular that peak intensities of ^29^Si MAS NMR spectra for various samples in the composition range 0.1 ≤ Al/(Al+Si) ≤ 0.5 could be best interpreted using the principle of homogeneous dispersion of charges (HDC). The principle is understood as a consequence of electrostatic interactions (Herrero *et al.*, 1986 ▸) and implies both Al–O–Al avoidance and homogeneous distribution of Al over the 6-rings of the sheet. These authors emphasized the need to consider charge interactions between two Al/Si sheets across the interlayer space. Ordering phase transitions in phyllosilicates 2:1 have also been studied through Monte Carlo methods (Palin *et al.*, 2001 ▸; Palin *et al.*, 2014 ▸). The latter authors determined in particular an ordering pattern of the tetrahedral sheet that was not considered by the former. Recently, we have proposed that the composition and Al/Si distribution in some ordered and disordered tectosilicates might obey the principle of maximal independence (Moreira de Oliveira Jr & Eon, 2024 ▸; Moreira de Oliveira Jr & Eon, 2025 ▸). At first sight, it would seem that Si MAS NMR data strongly invalidate the principle in the case of phyllosilicates. However, graph-theoretical models devised for tectosilicates cannot be applied directly to the study of micas since each triple layer forms a 2-periodic component of the respective net. The purpose of this work is to present a simple adaptation of the model, construct models for regularity in disordered phyllosilicates 2:1 and discuss their application to the analysis of peak intensities in Si NMR spectra of muscovite, phlogopite and vermiculite.

Sections 2 and 3 introduce graph-theoretical models that are used to describe Al/Si disorder in aluminosilicates. We analyse in particular the origin of the Al–O–Al avoidance rule and extend it to the principle of maximal independence. The failure of the principle as applied to single layers, or **hcb** sheets, in the case of phyllosilicates is described in Section 4 and a new model based on double **hcb** sheets is presented in Section 5. Application of this model requires knowledge of the minimum Al/(Al+Si) value in a maximal independent set of the quotient graph of the double **hcb** sheet: it is shown in Section 6 that this value is 1/4, which corresponds exactly to the ideal composition of muscovite. Section 7 proposes an interpretation of the topology of the **hcb** sheet as the Cayley colour graph of a multiplicative group with three generators; this description allows an easy determination of quotient graphs of **hcb** by translation subgroups of small index. The following part of the analysis showed that groups of index 4 provided satisfactory models of regularity in disordered phyllosilicates in the Al/(Al+Si) concentration range of interest. Application of the principle of maximal independence to the two quotient graphs of order 8 of **hcb** is then performed in Section 8, leading to models for disordered muscovite with the ideal composition. An analysis of real materials with Si/Al 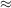 3 is given in Sections 9, 10 and 11 before concluding in Section 12.

## 2. Graph-theoretical tools

The main techniques used in this paper were developed in previous works by the authors; this section only intends to be a rough guide to the reader. Elementary concepts in graph theory are given by Harary (1972 ▸). Gross & Tucker (2001 ▸) provide an introduction to the concepts of voltage graph and Cayley colour graph. Infinite graphs now called nets, 2- or 3-periodic, were introduced in crystal chemistry by Wells (1977 ▸) to represent the topology of crystal structures. In particular, aluminosilicate frameworks are described as 4-connected 3-periodic nets, where vertices correspond to tetrahedral atoms and edges represent oxygen linkages or bridges between such atoms.

The labelled quotient graph *N*/*T* of a periodic net *N* with translation group *T* was introduced by Chung *et al.* (1984 ▸). It is a finite graph that preserves the degree (valency) of all vertices of the periodic net. It is constructed by identifying vertices and edges in *N* that are equivalent under translations in *T*. In other words, this implies taking vertex- and edge-lattices, respectively, as vertices and edges of the quotient *N*/*T*. In a labelled quotient graph, lattice vectors are attributed to edges. This requires specifying an origin 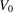 for every vertex-lattice *V* and indexing the respective vertices as 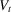, when 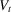 is the image of 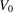 by 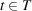. If vertex 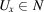 is adjacent to vertex 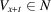, for every 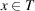, the two vertices *U* and *V* are linked by an edge *UV* in the quotient graph, and this edge is assigned the label (voltage) 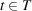.

An independent set *S* of vertices in a graph *G* is a subset of vertices inducing an *empty* subgraph, that is: there is no edge in *G* linking vertices in *S*. The independent set of vertices *S* is said to be maximal if the induced subgraph is no more empty after adding any new vertex from *G* to *S*. An independent set of vertices is called maximum when it has maximum cardinality among all independent sets of *G*. The lower and upper bounds of the cardinality of maximal independent sets are known, respectively, as the independent domination number and the independence number.

The well known Löwenstein rule (Löwenstein, 1954 ▸) admits a simple interpretation in graph-theoretical terms: it just says that Al atoms in aluminosilicates form an independent set of the respective 3-periodic net. We showed in previous works that the rule can be strengthened to a principle of maximal independence, shedding more light on the structure and composition of ordered and disordered materials. Specifically, we introduced the concepts of alveolus and hypergraph of correlations (Moreira de Oliveira Jr & Eon, 2025 ▸). An alveolus is the pre-image in the net of a subgraph induced (in the quotient graph) by the symmetric difference between maximal independent sets. Alveoli are substructures allowing Al/Si disorder in microscopic domains with a minimum local alteration of Al/Si order; each alveolus is associated with some hyperedge of the hypergraph of correlations. We recall that *regularity* was defined in the latter work as the repetition through the whole structure of a small number of patterns positioned according to a weak rule of order, *i.e.* a rule of order allowing for more than one neighbourhood for its members. The rule of order describing the Al/Si distribution in disordered materials is represented by the hypergraph of correlations. A detailed, step-by-step algorithm was described in the above-mentioned work, enabling the determination of alveoli in 2- or 3-periodic nets. An explicit construction of alveoli in phyllosilicates 2:1 can be found in the supporting information. In all our examples configurations were obtained using a home-made code.

## 3. Avoidance rules in 4-coordinated aluminosilicates

We claimed in a previous paper (Moreira de Oliveira Jr & Eon, 2024 ▸) that the Al–Al avoidance, or Löwenstein rule, in aluminosilicates cannot be imputed to a particular property of Al but is the consequence of Si–Si avoidance. We briefly recall the argument. In the case of a finite 4-coordinated graph the number of Si–Si and Al–Al links is related through the equation |Si–Si| + 2|Al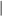 = |Al–Al| + 2|Si|, where |*X*| represents the number of *X* species, atoms or links, in the graph. According to the fourth rule of Pauling (1929 ▸), ‘in a crystal containing different cations, those of high valency and small coordination number tend not to share polyhedron elements with one another’. That rule implies minimizing the number of |Si–Si| links in aluminosilicates; but in a crystal with given |Si| and |Al| values, this result can only be obtained by minimizing the number of |Al–Al| links, as the above equation shows. If, moreover, |Si| ≥ |Al|, the minimum |Si–Si| value is given by |Al–Al| = 0.

The origin of the Al–Al avoidance rule has therefore been ascribed to electrostatic interaction. The rule implies minimizing the strongest repulsion between first neighbours. But it is not very restrictive and there is still room for other conditions. In particular, we may ask to minimize repulsion between next-to-nearest-neighbour atoms. Let us define 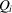 species as Si species of the type Si(OAl)_*i*_(OSi)_4−*i*_ (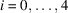) in a 4-coordinated framework. Obviously 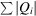 = |Si|, with summation over the index *i*. If we admit avoidance of |Al–Al| links, we also get 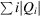 = 4|Al|. Summing up over all 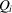 species, it easily follows that |Si–Si–Si| = 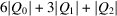. Note that |Si–Al–Si| = 6|Al| is defined by the composition of the crystal; hence minimizing next-to-nearest-neighbour repulsions amounts to minimizing |Si–Si–Si|, which given the factor 6 is most probably reached for 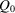 = 0. But we also find |Al–Si–Al| = 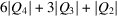, leading to |Si–Si–Si| + 12|Al| = |Al–Si–Al| + 6|Si|. The latter relation shows that, for a given composition, the numbers |Si–Si–Si| and |Al–Si–Al| increase or decrease together as a function of the Al/Si distribution. This would provide a justification to Dempsey’s criterion (Dempsey, 1968 ▸).

We can get more insight by considering the fifth rule of Pauling (1929 ▸), also called the rule of parsimony. This rule states that ‘the number of different types of constituents (coordination polyhedra, atomic sites) in a stable crystal tends to be small’. Applied to aluminosilicates, this suggests that all Si atoms tend to have the same environment, ideally all belonging to the same 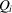 type. For example, applying the two previous relations to compounds with |Si| = 4|Al| and |Si| = 2|Al|, we would expect peaks at 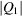 = |Si| and 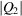 = |Si|, respectively. For ideal muscovite assumed to be 4-coordinated (see below) with |Si| = 3|Al|, we would expect 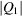 = 2|Al| and 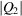 = |Al|. In any compound with 2|Al| ≤ |Si| ≤ 4|Al|, we would expect 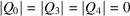. This discussion can be summarized in the following statement.

Principle 3.1 (Maximal independence) Ordered or disordered (*M*, Si) (*M* = Al, Be, …) distributions in tectosilicates tend to correspond to maximal independent vertex sets of the periodic net attached to the respective framework.

For compounds with Al content larger than the independent domination (ID) number, we expect 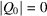. Conversely, when the Al content is lower than the ID number, we expect that stable compounds are those with the lowest 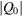 value.

## 4. Avoidance rule in phyllosilicates

A similar argument can be applied to Si in phyllosilicates, assuming instead that the atom has effective coordination 3 in the Al/Si sheet. The two balance relations are now 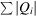 = |Si| and, if we admit avoidance of Al–Al links, 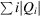 = 3|Al| with summation over the index 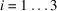. According to the rule of parsimony, we expect 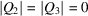 for |Si| ≥ 3 |Al|. We can thus calculate theoretical values for 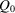 and 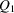 concentrations using the two balance equations. The results are compared in Table 1 ▸ with experimental data published by Herrero *et al.* (1989 ▸).

It appears that the model is in accordance with experimental values at low Al content but shows significant deviation with increasing Al content. In particular, the model is quite inadequate for Si/Al = 3, where the principle of maximal independence seems to fail. [The Al/(Al+Si) ratio of maximal independent sets in the **hcb** layer varies from 0.25 to 0.5.] Note that completely different sets of values are mathematically admissible since we have two equations and four variables. Herrero *et al.* (1986 ▸) have shown, however, that electrostatic interactions between opposite sheets cannot be overlooked. This is why we propose to apply the principle of maximal independence to the double **hcb** layer.

## 5. The model

In order to apply the principle of maximal independence, we need a connected graph. We begin with a comparative analysis of the structures of muscovite with ideal formula KAl_2_[AlSi_3_O_10_](OH)_2_ and space group 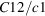 (Catti *et al.*, 1994 ▸) and margarite with ideal formula CaAl_2_[Al_2_Si_2_O_10_](OH)_2_ and space group 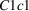 (Joswig *et al.*, 1983 ▸).

As shown in Fig. 1 ▸ the structure of muscovite is formed by parallel T–O–T layers containing a central *octahedral* (O) sheet of Al between two *tetrahedral* (T) sheets of Al and Si. Cations are located in the interlayer space. The structure of every T sheet is described by the honeycomb (**hcb**) net. The distance between the two intralayer T planes marked as (a) and (b) in the figure is about 5.45 Å, significantly larger than the distance of 4.08 Å between T planes marked as (b) and (c) across the interlayer space. This indicates a larger interaction between T sheets across the interlayer space in comparison with T sheets from the same layer.

The structure of margarite follows essentially the same alternation of T–O–T layers with cations in the interlayer space. Fig. 2 ▸ shows two projections of the two Al/Si sheets sandwiching the cation interlayer, a two-layer 2-periodic structure that we shall call a *sandwiching network*. These images (Figs. 1 ▸ and 2 ▸) were obtained using the program package *TOPOSPRO* (Blatov *et al.*, 2014 ▸).

For the sake of clarity, Fig. 2 ▸(*a*) shows a projection along the *c* axis, which is slightly tilted in relation to the normal to the (001) plane, along [103]. The latter projection, in Fig. 2 ▸(*b*), shows that the symmetry of tetrahedral (Si/Al) sites in the T sheet is virtually 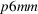, the plane group of the honeycomb net. It is perhaps significant that the two Al/Si layers appear to respect a rigorous alternation of Al and Si not only within each T sheet but also in the direction of the normal to the plane: across the interlayer space and opposite to Si only Al atoms are found, and conversely. This observation suggests that electrostatic interactions across the interlayer space are indeed strong enough to correlate the Al/Si distribution in the two sheets. Since they have a similar effect to the avoidance rule, and probably the same origin, we could represent them by adding an edge joining the pairs of atoms across the interlayer space. We emphasize that the resulting 2-periodic net is 4-coordinated.

Topologically, the Al/Si sheet may be represented by the 2-periodic net **hcb** (the honeycomb net) with labelled quotient graph drawn in Fig. 3 ▸.

We can thus represent the sandwiching network (the double Al/Si sheet across the interlayer space) by joining two equivalent quotient graphs as in the bottom-left part of the figure. Grey edges represent direct electrostatic interaction through empty space between opposite ions while black, blue and red edges indicate an oxygen bridge. The independence ratio of both quotient graphs is 1/2; filling the pre-image of the red vertices with Al, for instance, generates the sandwiching structure of margarite. But we will use a simpler representation with a partial 2-colouring as given on the bottom right of the figure, where every vertex corresponds to a pair of opposite atoms across the interlayer space. Here we use two colours, say blue and red, to distinguish two independent subsets, one per sheet, associated to Al atoms; black vertices represent Si atoms. In spite of the hidden edge, there is no ambiguity in the representation since only one of the end-vertices can belong to an independent set *S*. Hence a black vertex indicates that neither end-vertex belongs to *S* while a coloured (non-black) vertex shows which of the two end-vertices belongs to *S*. Under these conventions, a partial 2-colouring describes an independent set whenever two linked vertices from *S* have received different colours. The set is maximal when every black vertex has at least one neighbour of each colour. The minimum number of Al atoms in such a configuration is known in graph theory as the 2-rainbow ID number. The problem is NP-complete and has no general solution but it was possible to solve it here, due to the special properties of the **hcb** net. Maximal independent sets of the double **hcb** layer can also be interpreted as T_4_-colourings of the **hcb** net, as discussed in Appendix *A*[App appa].

## 6. The 2-rainbow ID number of quotient graphs of **hcb**

We start with an analysis of the possible configurations of 6-rings in an arbitrary quotient graph of the **hcb** net, as shown in Figs. 4 ▸, 5 ▸ and 6 ▸. We distinguish eight non-equivalent configurations based on the number of Si atoms, say *s*, and Si–Si links, say *p*, per ring, which we note 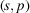; the number of each kind of rings with a given configuration associated to some independent set is written 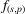.

Some of these configurations deserve attention, in particular in relation to the sub-configuration of 3-vertex Al–Si–Al paths. We will say that an Al–Si–Al path is symmetrical if both Al have the same colour. Thus, alternation of Al of different colour in rings 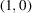 forces a symmetrical configuration of the Al–Si–Al path running along such a ring, which prevents participation of the Si atom in a Si–Si link if maximality conditions must hold. There are different possible choices of colour for Al in the two rings of type 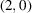, some of them necessarily associated with the presence of isolated Si atoms; Fig. 5 ▸ only shows configurations where both Si atoms can participate in Si–Si links. Rings of type 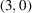 include at least one isolated Si, as shown in Fig. 6 ▸.

We shall write different balances for faces, edges and vertices of the quotient graph. Call, respectively, *F*, *P* and *S* the number of faces, Si–Si links and Si atoms in the quotient. We will also distinguish 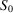 and 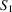, the number of isolated and paired Si atoms, respectively. The three balances are given as follows, where the sum is over the whole set of rings (or faces) in the quotient graph:
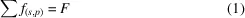

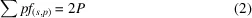

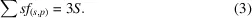
The factors 2 and 3 in equations (2) and (3) correspond to sharing of edges and vertices between two and three rings, respectively. We can then determine 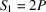 and 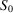 = 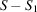: 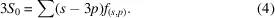 The respective expression is reported as the first row in Table 2 ▸.

Call *R* the ring subset whose elements are those rings of type 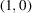 and 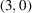.

Lemma 6.1 There is an injective mapping from *R* to the set of isolated Si atoms.

Proof Isolated Si atoms are in one-to-one correspondence with symmetrical Al–Si–Al paths since (i) a single unsymmetrical Al–Si–Al path is centred at any paired Si atom and (ii) out of the three Al–Si–Al paths centred at an isolated Si, one and only one is symmetrical. On the other hand, we know that each ring in *R* contains at least one symmetrical Al–Si–Al path. We can then map each ring 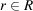 to some symmetrical Al–Si–Al path running along the respective ring *r*. This mapping is clearly injective since, in contrast with edges and vertices, 3-vertex paths are not shared between adjacent rings. Using the composition of this mapping with the one-to-one correspondence between symmetrical Al–Si–Al paths and isolated Si atoms, we can build an injective mapping from *R* to the set of isolated Si atoms.

□

As a consequence of the lemma we get 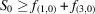 or 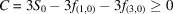.

The second row in Table 2 ▸ develops the expression of *C*, allowing the inequality 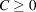 to be rewritten as
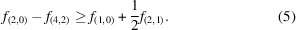
Upon inserting this inequality in the expression of 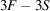, we finally find
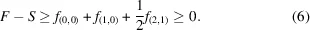
For quotient graphs with 2*n* vertices, we have *F* = *n*, so that 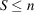: the 2-rainbow ID number of any quotient graph of **hcb** is thus 1/2. This limit is actually reached for 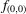 = 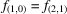 = 0, as shown below. It follows that the ID number of the quotient graph of the double **hcb** layer is 1/4. For such sets of the single **hcb** layer, we must also have [summing over meta- and para-(2,0)] 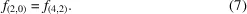


## 7. **hcb** and its quotient graphs

We need to be able to easily find out possible quotient graphs of the net **hcb**. To this end, it is useful to consider the net as the Cayley graph of the group 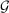 = 〈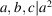 = 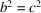 = 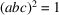
〉, that is the infinite multiplicative group generated by the three generators 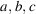 satisfying the four given relations. One of us (Eon, 2018 ▸) has already shown that uninodal 2-periodic nets are isomorphic to Cayley colour graphs of plane groups [see also Baburin (2026 ▸)]. The above definition of generators and relators is not associated to symmetry operations but instead to properties of the net **hcb**. Each generator corresponds to an edge of the quotient graph; the last relator describes a strong ring in **hcb**.

Proposition 7.1 The Cayley graph of the group 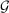 is isomorphic to the net **hcb**.

Proof By construction, the vertices of the Cayley graph correspond to the elements of the group. As shown in Fig. 7 ▸, a vertex *x* is linked to the three vertices *xa*, *xb* and *xc* through the edges of colours *a*, *b* and *c*, respectively. Observe that the fourth relation implies equivalently 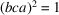 (multiply, respectively, by *a* on the left and on the right of both sides of the equation) and 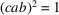 (multiplying this time by *c*). Thus, the fourth relation ensures the existence of three hexagonal cycles at every vertex (see the vertex 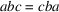 in Fig. 7 ▸).

The subgroup generated by the three products 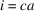, 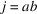 and 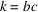 is abelian, as easily verified. For example, 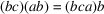 = 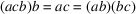. Since their inverse are, respectively, *ac*, *ba* and 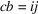, it follows that any product of an even number of elements of 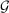 can be written as 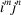 for some 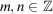 and hence that any product of an odd number of elements can be written as 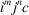. This tells us that the Cayley graph is bipartite, as expected, and admits the free abelian group 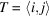 as translation group with actions defined by 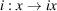 and 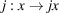, where an origin must be first defined. Observe that setting the origin at any vertex of the other part of the bipartition inverses both *i* and *j*. These operations are naturally vertex and edge colour-preserving and the quotient of the Cayley graph by *T* is isomorphic to 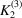.

□

It is clear that quotient graphs of the net **hcb** are finite, cubic, bipartite graphs. Let 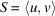 be a translation subgroup of *T*; then the respective quotient graph of **hcb** is the Cayley colour graph of the quotient group 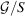. As such it must satisfy the same relations defining 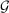 together with new relations associated to the quotient 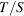. In fact, the quotient group 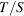 acts as a group of colour-preserving automorphisms in 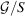 that act transitively on any part of the bipartition. The converse is true:

Proposition 7.2 Any finite, cubic, bipartite graph *G* that (i) can be coloured as above, (ii) satisfies the relation 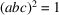 and (iii) admits a colour-preserving automorphism subgroup *A* acting transitively on both parts of the bipartition is a quotient graph of **hcb**.

Proof The group *A* acts freely on *G*. Let 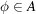 and *v* such that 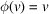; since ϕ maps edges to edges of the same colour, the three incident edges at *v* are fixed and so are the three vertices adjacent to *v*. By induction, the whole (connected) graph is fixed and ϕ is the identity. Again, since the automorphisms in *A* preserve colours, they preserve the voltages induced by these colours over any cycle of *G*. These two properties show that *A* corresponds to translations of the derived net. Then 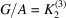 with three voltages *a*, *b* and *c* satisfying the relation 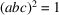 is the quotient graph of **hcb** by its full translation group, showing that *G* is a quotient graph of **hcb**.

□

Corollary 7.1 Any two 2-colour cycles of the same colours have the same even length.

Proof Because the group *A* is transitive on each part of the bipartition and maps a 2-colour cycle to a 2-colour cycle of the same colours, any two cycles of the same two colours must have the same length. This length must be even since the graph is bipartite.

□

The following properties are also worth noting.

Proposition 7.3 2-Colour cycles of the same colours do not intersect and any 2-colour cycles of different colours intersect at a single edge.

Proof Consider a blue–green walk *w* crossing vertex 1 (see Fig. 7 ▸). The sequence of vertices along *w* is necessarily 1, *c*, *ca*, *cacetc*., which form the set 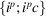 for 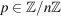: because *G* is finite *w* is a cycle of length 2*n*. By construction, *w* is the only such cycle; notice that *w* is invariant by translation *i*. Since *A* is transitive over the two parts of the bipartition and preserves edge colours, it is also transitive over the edges of any colour. Hence a single blue–green cycle can cross any blue or green edge. Take now the red–blue cycle 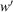 containing edge *e*; the same argument shows that the set 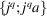 for some 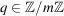 determines a cycle of length 2*m*. Both cycles *w* and 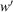 share the blue edge *e* at vertex 1. Suppose another blue edge 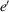 is shared by these two cycles. This edge can be obtained from *e* by some translation, say 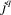 along the red–blue cycle. The translated cycle 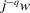 is thus also a blue–green cycle containing edge *e*, but by uniqueness, 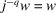 meaning that 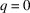 modulo *m* and 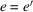. Consider now a pair of blue–green and red–blue cycles obtained, respectively, by translation 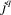 and 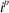 from *w* and 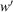. Both cycles contain the edge 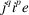.

□

These two properties enable an easy analysis of possible quotient graphs of **hcb**. Let us consider some simple examples, all excluding the presence of multiple edges. First, the graph 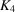 which is not bipartite cannot be a quotient graph of **hcb**. Next, we want to determine possible quotient graphs of order 6. Delete all green edges, for instance; what remains is a subgraph of degree 2, that is: a set of blue–red cycles. In accordance with Corollary 7.1[Statement corollary7.1], the six vertices form a single 6-cycle. Since there are no odd cycles, we can only add green edges, as indicated in Fig. 8 ▸. It can now be checked that all conditions are satisfied so that **hcb** shows a unique quotient graph by a translation subgroup of index 3, up to isomorphism.

We can now determine the respective translation subgroup by looking at cyclic conditions associated to 2- or 3-colour cycles. Choosing 4-cycles (the shortest cycles), we find for example the words *caba* and *abcb* corresponding, respectively, to translations 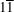 and 12 of **hcb**, so that 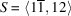.

Consider now quotient graphs of order 8 and the set of their 2-colour cycles; according to Corollary 7.1[Statement corollary7.1] each set contains necessarily two 4-cycles or one 8-cycle. The two possible cases, up to isomorphism, are represented in Fig. 9 ▸. On the left there is one blue–green and one red–green 8-cycle; red–blue 2-colour cycles are 4-cycles. We can take as generators of the subgroup the words *cacb* and *abab* associated to translations 21 and 02 so that 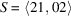, corresponding to a rectangular unit cell. On the right, there are only 4-cycles associated to translations 20, 02 and 22, so that we can choose 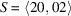, corresponding to a hexagonal unit cell.

It is worth noting that Palin *et al.* (2001 ▸) used the rectangular unit cell to evaluate exchange interactions in the **hcb** sheet. In contrast, they described the ordered pattern in muscovite through the hexagonal unit cell.

## 8. The structure of ideal muscovite

The sandwiching net in the disordered structure of muscovite, with ideal formula KAl_2_[AlSi_3_O_10_](OH)_2_ and space group 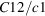, can be described by the same labelled quotient graph shown in Fig. 3 ▸. It is clear, however, that this graph admits a single maximal independent set with alternation of Al of different colour; according to the model we have been developing, this unique set describes the ordered structure of margarite. We must then take the quotient of the **hcb** net by translation subgroups of higher index if we want to find ordered structures modelling the ideal Si/Al ratio of 3. Only two graphs, respectively, of order 4 and 6 may correspond to quotient graphs of **hcb** by subgroups of index 2 and 3: the cycle 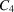 for the subgroup 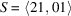 with two double edges and 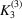 for the subgroup 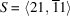, as shown in Fig. 8 ▸. In both cases, there is a unique configuration, up to isomorphism, with Si/Al = 3 leading necessarily to an ordered structure. Fig. 10 ▸ shows a 2-colouring of each graph where Al atoms are in the meta position according to the nomenclature adopted by Herrero *et al.* (1986 ▸). In the rectangular unit cell (subgroup of index 2) the distribution is homogeneous between the two sides of the sheet; this is also the first electrostatically most stable configuration determined by Herrero *et al.* (1986 ▸). Although the deficit of charge in this distribution is homogeneously balanced over every hexagonal ring, it does not reproduce the peak intensities of 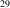
Si MAS NMR spectra, as published by Herrero *et al.* (1987 ▸).

In fact, NMR resonance is sensitive to electronic density at the nucleus, which is differently affected by through-space electrostatic interaction and chemical bonding. We will denote 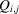 those 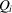 species, that is Si species of the type Si(OAl)_*i*_(OSi)_3−*i*_ (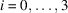), all atoms being in the same sheet, in electrostatic interaction with *j* Al atoms (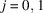) through the cation interlayer. The distributions shown in Fig. 10 ▸ can then be described as 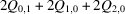 per unit cell in the rectangular case and 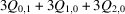 in the hexagonal unit cell. The observed Si NMR spectrum depends on the relative strength of the two interactions; we will consider three extreme situations, analysing the case of the rectangular cell. (i) If electrostatic interactions dominate, the spectrum is given only by the *j* value and corresponds to 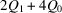. (ii) If both interactions are similar, the spectrum is given by the sum 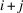 and corresponds to 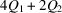. (iii) If chemical bonding dominates, as is more probable considering the Si–Si distance across the cation interlayer, namely about 4.5 Å against 3 Å in the sheet, the spectrum is given by the *i* value and we expect three resonances with equal intensity, close to 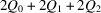 where electrostatic interaction might determine a small perturbation to purely chemical bonding values (intensities and chemical shift). The observed spectrum is, however, still rather far from the latter expectation. We must then refer to subgroups of index 4, the lowest index allowing the existence of a disordered Al/Si distribution.

We saw in Section 7 that the two acceptable subgroups of index 4 determine the same quotient graph, the graph of the cube. This presents 24 2-colourings (see Appendix *B*[App appb]), that is 24 maximal independent sets of the double **hcb** layer with Si/Al = 3, 12 that can be described as 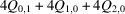 and 12 as 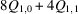. Of course, the Al/Si distribution in the unit cell depends on the respective subgroup. Because 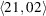 is a subgroup of 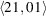, we also get four configurations in the rectangular unit cell that are isomorphic to that shown in Fig. 10 ▸ (left). Figs. 11 ▸ and 12 ▸ show, respectively, five different configurations, up to isomorphism: (i) three in the rectangular unit cell and (ii) two in the hexagonal one. The 12 configurations isomorphic to that drawn in Fig. 12 ▸(*a*) are also isomorphic (through rotation) to the four above-mentioned already seen in Fig. 10 ▸ (left). The four remaining kinds of configurations can be described as (i) Al in the meta or para position and (ii) homogeneous or inhomogeneous density of charge. It is worth noting (i) that Fig. 11 ▸(*c*) corresponds to the second electrostatically most stable configuration obtained by Herrero *et al.* (1986 ▸), (ii) that configurations in Figs. 11 ▸(*a*), 11 ▸(*c*) and 12 ▸(*a*) present homogeneous density of charge, and (iii) that configurations in Figs. 11 ▸(*c*) and 12 ▸(*b*) present only Al in the para position in each sheet. Moreover, the structure associated to a single layer (blue or red) in Fig. 12 ▸(*b*) corresponds to the configuration of the ordered sheet found by Palin *et al.* (2001 ▸) with four possible configurations in each sheet. The 12 configurations (three available configurations in one sheet for each configuration in the other sheet across the interlayer), similar to Fig. 12 ▸(*b*), correspond to stacking of ordered sheets as proposed by these authors. Notice that they only mentioned eight possibilities for stacking. We are, however, looking for regularity in disordered double layers, implying that those 24 configurations are instead local patterns repeating themselves in each double layer. In a disordered material with a random distribution of these configurations, one may expect that the Si NMR spectrum corresponds to the average, given as 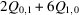

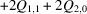
. Assuming that the spectrum is defined by the first index, that is, by chemical bonding, the expected spectrum would be 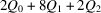, in reasonable agreement with experimental data.

In order to model regularity in disordered Al/Si distributions related to those configurations, we apply the methodology developed in our previous paper (Moreira de Oliveira Jr & Eon, 2025 ▸). We have indeed observed that small subsets of maximal independent sets may overlap; in this case the respective configurations may be combined randomly with minimal changes in one or two crystallographic directions, forming disordered, maximal independent micro-domains. We have also shown that homogeneous Al/Si distributions are obtained for transitive nets, which is the case of **hcb**. Compare for instance the two configurations in Figs. 11 ▸(*a*) and 11 ▸(*c*). Only the right part of the central hexagonal ring is different. In fact, the four unshared Al atoms belong to another hexagonal ring running over the two **hcb** sheets and each pair constitutes a maximal independent set of diametrally opposed vertices in this ring. Recall that 6-cycles have three 2-vertex and two 3-vertex maximal independent sets. This leaves room for another 2-vertex and for two 3-vertex maximal independent sets on the same ring. Because this ring has voltage 10, its pre-image in the double **hcb** net, the alveolus, is an infinite line that can be filled by one Al every three sites in three different ways or by one Al every two sites in two different ways. 2-Vertex subsets lead to disorder in the respective direction while 3-vertex subsets add non-stoichiometry.

Fig. 13 ▸ shows (in brown) three alveoli running along the 10 axis, associated to a 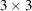 fragment of a **hcb** sheet. The right part reproduces three times, along the 10 axis, the configuration given in Fig. 11 ▸(*a*). The left part reproduces a second configuration obtained by filling differently the alveolus, also with one Al every three sites. The Al/Si sequence along the middle alveolus starts as that on the left part and ends as that on the right part; alteration of the sequence generates non-stoichiometry in the central cell, with an increase in the Al concentration.

The complete analysis of overlapping configurations with maximum overlap is summarized in the hypergraph of correlations shown in Fig. 14 ▸ for the rectangular unit cell (Fig. 10 ▸). The graph is connected and all hyperedges are triangles, describing alveoli parallel to axes 10 or 01, all pre-images of a 6-cycle in the quotient graph. The fragment in Fig. 13 ▸ was constructed from configurations R05 and R04 (appearing, respectively, on the left and on the right of the figure), forming a hyperedge together with configuration R02 in direction 10. A full description of all the configurations is given in the supporting information.

A different method of construction of a 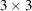 disordered fragment of the **hcb** double layer was used in Fig. 15 ▸, as represented in matrix form below:
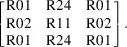
In this fragment the four configurations are shared pairwise by four hyperedges. For instance, R01 and R02 belong to the hyperedge {R01, R02, R03} oriented in the 01 direction. This means that each configuration, R01 and R02, can form infinite rows running along 01, one next to the other in direction 10. The respective alternating blue–red square induced in the hypergraph by these four configurations allows then a distribution symbolized by the above matrix. Visually, the method generates more disorder than the previous one, with a more even distribution of ring configurations (3, 1) and (3, 0). In this example, only the most electrostatically stable configurations (those with homogeneous density of charge) were used, which amounts to using the subgraph of the hypergraph induced by the white and green vertices, equivalent to R01 and R11, respectively. The expected Si NMR spectrum of a random distribution of these 20 configurations is given by the average over individual spectra 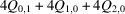 and 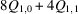 with weight 12 and 8, respectively. Considering only the first index, we expect the experimental spectrum to be close to 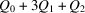, in reasonable agreement with data in Table 1 ▸ for Al/(Al+Si) = 26%.

Fig. 16 ▸ shows the hypergraph of configurations for the hexagonal unit cell of the double **hcb** layer. See the supporting information for a description of the configurations. Again, all hyperedges are triangles associated to 6-cycles of the quotient graph. We note, however, the presence of alveoli along the three axes of the hexagonal cell, 10, 01 and 11, as well as finite alveoli in the form of 6-cycles. As in the rectangular case, these allow disorder and non-stoichiometry. Interestingly, finite alveoli can create two-dimensional disorder. An example is given in Fig. 17 ▸: here blue and black vertices are ordered, only red vertices are randomly distributed as 2-vertex maximal independent sets over a periodic subset of the hexagonal faces of the **hcb** layer, exactly one per unit cell. Non-stoichiometry can be generated by filling a 6-cycle with three Al, as shown in the figure for the marked hexagonal face.

## 9. Real materials with Si/Al ≈ 3

Consider first the case 20% ≤ Al/(Al+Si) < 25%. Such materials are not saturated, meaning that there are necessarily a few 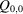 species. According to the principle of maximal independence, we expect the respective concentration to be the lowest possible. Using the same rectangular and hexagonal unit cells as before, with *Z* = 4, the Al content per unit cell is approximately in the interval 3 ≤ Al < 4, thus one Al less than the ID number. Since the respective configurations should be as little unsaturated as possible, we conclude that adding at most one Al atom per unit cell should provide saturation. In other words, these configurations can be derived by withdrawing randomly, here and there in the layer, a single Al atom per unit cell. Working out the 24 2-colourings of the quotient graph given in Fig. 9 ▸, we derive 48 minimally un­saturated configurations with three Al per unit cell with spectrum 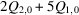

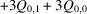
 and 48 with spectrum 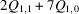

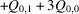
. (We discarded 112 configurations each containing five or six 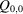 species.) The average spectrum over the remaining 96 configurations is thus 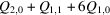

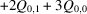
, so that we expect the experimental spectrum to be close to 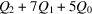 (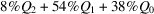) for samples with Al/(Al+Si) = 3/16 ≈ 19%. This is in good agreement with data in Table 1 ▸ for Al/(Al+Si) = 20%.

Muscovite, with Al/(Al+Si) ≈ 22%, is necessarily unsaturated but an average of only one Al atom every two unit cells is missing in relation to maximal independent configurations corresponding to the ID number. We might thus expect the spectrum to represent the average between the 24 respective 2-colourings of the quotient graph and the 96 deficient above-described 2-colourings, with equal weight for both kinds of configurations, that is 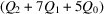

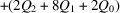
 = 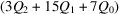, approximately 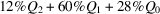. This is in nice agreement with the observed spectrum reported by Herrero *et al.* (1989 ▸): 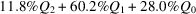. It is worth noting that using instead the 20 2-colourings as shown in Figs. 11 ▸(*a*) and 11 ▸(*c*) does not yield so good a result. This observation suggests that the density of charge is probably not homogeneous.

## 10. Synthetic samples with low Al content

Analysis of the quotient graph shown in Fig. 9 ▸ yields 40 minimally unsaturated configurations with two Al per unit cell with spectrum 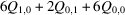 so that we expect the experimental Si NMR spectrum to be 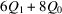, close to 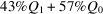 for Al/(Al+Si) = 12.5%. Considering that the Al concentration is in fact slightly lower, the agreement with the experimental spectrum of the first sample with 11.5% Al is rather good. Indeed, for a single Al atom per unit cell, the spectrum is 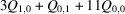 so that the experimental spectrum should be 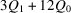, close to 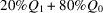. A weighted average corresponding to Al/(Al+Si) = 11.5% (16% unit cells with 1 Al and 84% with 2 Al) would give the observed spectrum: 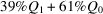.

A weighted average corresponding to Al/(Al+Si) = 15% (60% unit cells with 2 Al and 40% with 3 Al) should give the spectrum 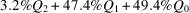, again in good agreement with experimental data.

## 11. Phlogopite and vermiculite

We have seen that alveoli associated with configurations of cardinality 4 of the quotient graph in Fig. 9 ▸ are all derived from 6-cycles of the quotient graph, with two Al per cycle. These same 6-cycles also admit two configurations with three Al: this is the origin of configurations of cardinality 5. In fact, every alveolus can be a source of non-stoichiometry and disorder, resulting in disordered samples with any possible Al concentration in the range 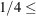 Al/(Al+Si) 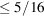. Both phlogopite and vermiculite belong to this domain. In particular, the composition of vermiculite is close to the upper limit. For ideal samples with Al/(Al+Si) = 5/16, we expect the spectrum to be 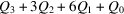, close to 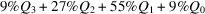, in reasonable agreement with experimental data given for Al/(Al+Si) = 30.5% by Herrero *et al.* (1989 ▸).

## 12. Final observations

We reported here a detailed application of the principle of maximal independence to the analysis of disorder in phyllosilicates 2:1. Specifically rectangular and hexagonal unit cells of **hcb** with *Z* = 4 provided each 24 local configurations forming the vertex-set of the respective hypergraph of correlations. The different patterns considered by previous authors (Palin *et al.*, 2001 ▸; Herrero *et al.*, 1989 ▸) were found as a subset of these 48 configurations. Regular patterns in the Al/Si distribution in phyllosilicates 2:1 can be derived from the hyperedges of the hypergraph of correlations. As an illustration, we have built a few models of regular Al/Si distributions for stoichiometric and non-stoichiometric layers. Averaging over Si neighbourhoods in the whole sets of configurations yielded theoretical values for intensities of 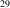
Si MAS NMR spectra that are in good agreement with experimental intensities for all materials analysed. Our analysis suggests that distributions with homogeneous density of charge and Al in the para position are more stable, supporting the conclusions of the above-mentioned authors. Our results clearly indicate decreasing values for 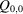 [Si(Si_4_)] species, converging to zero for the last material reported in Table 1 ▸, where we expect saturation (maximal independence) at Al/(Al+Si) ≈ 25%. These results also clearly show that through-space electrostatic interaction between opposite sheets across the cation interlayer strongly contributes to the stability of the material and must be included in the model, as suggested by Herrero *et al.* (1989 ▸). Si–Si interaction across the interlayer space should be avoided in the same way as Si–O–Si links in the layer.

It is hoped that the analysis performed on a simple 2-periodic net might help to explain the methodology as well as demonstrate its efficiency, since it can be applied to any aluminosilicate. We recall that the method was introduced in a previous work (Moreira de Oliveira Jr & Eon, 2025 ▸) where a detailed step-by-step algorithm was also fully described.

It might seem surprising that such a simple model as derived from the principle of maximal independence could predict with some precision the peak intensities in a Si NMR spectrum, and hence the Al/Si distribution in disordered materials. In fact, the Si–O–Si avoidance rule has its roots in the foundations of electrostatic theory. In an ionic model of the crystal, where all ions present in some crystalline domain must be considered, it is clear that the avoidance rule leads to minimizing the strongest repulsions by dispersing the highest charges. Since the strongest interactions correspond to first and second neighbour interactions [see 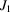 and 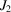 in Palin *et al.* (2001 ▸)] models based on concepts of independence offer a qualitative, but efficient and easy treatment of electrostatic interactions.

## Supplementary Material

This material explains the construction of the correlation hypergraphs associated with the two labeled quotient graphs of order 8 of hcb. DOI: 10.1107/S2053273326009794/ae5187sup1.pdf

## Figures and Tables

**Figure 1:**
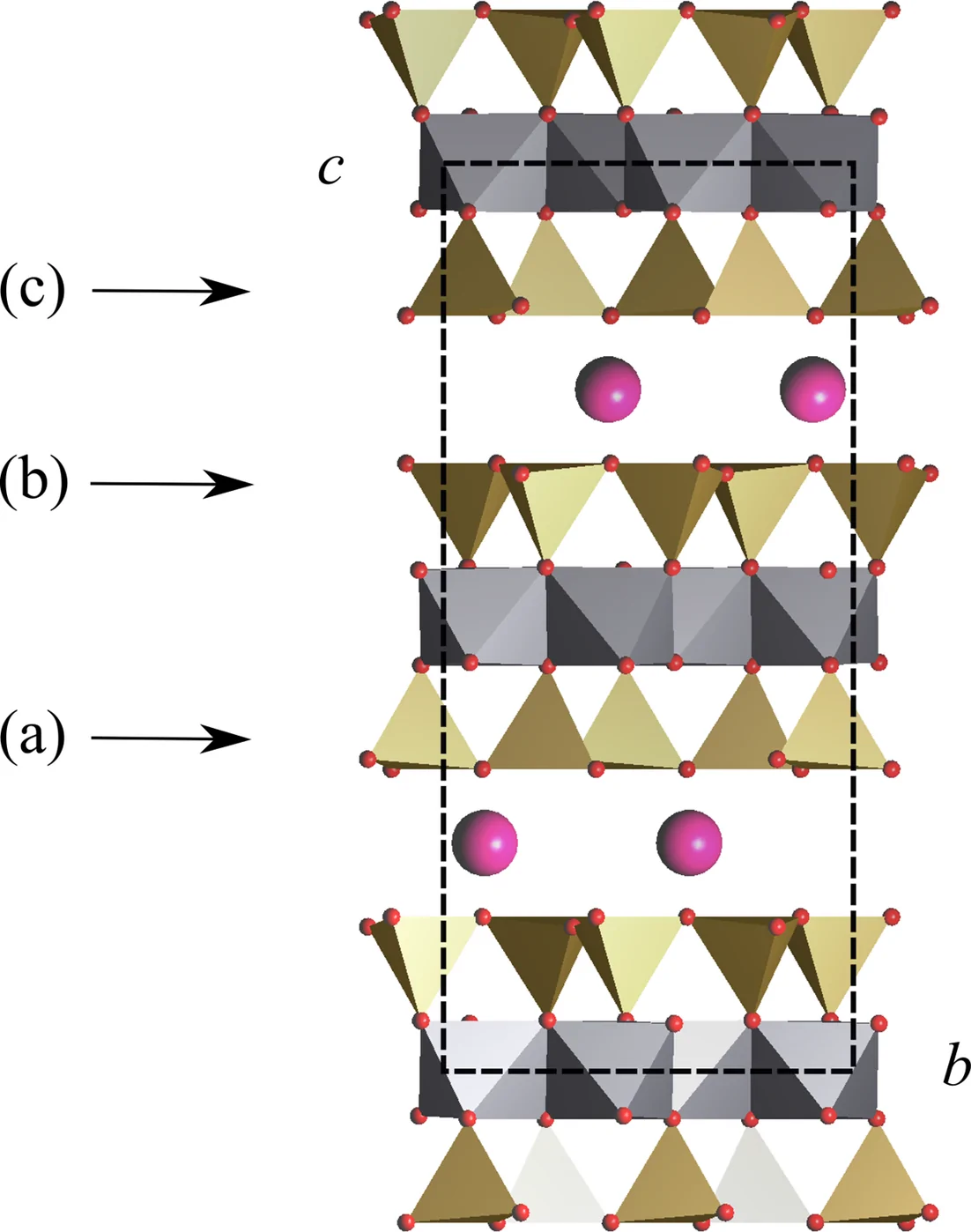
View along [100] of the idealized structure of muscovite showing the T–O–T layer and cation interlayer: Si (yellow), Al (grey), O (red) and K (magenta).

**Figure 2:**
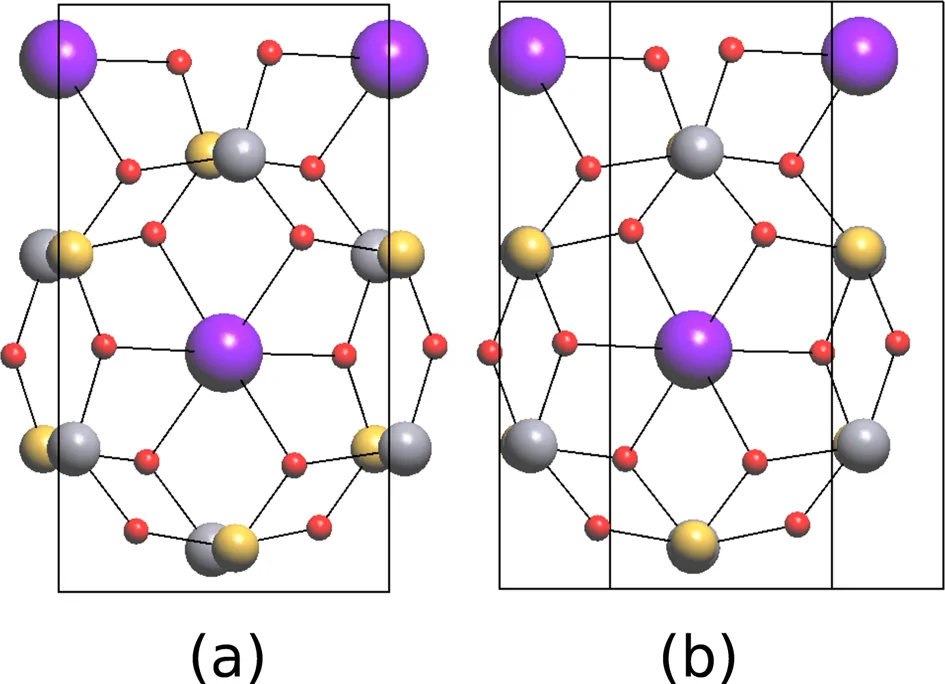
View along (*a*) [001] and (*b*) [103] of the interlayer cations with the two adjacent Si/Al sheets in margarite: Si (yellow), Al (grey), O (red) and Ca (purple).

**Figure 3:**
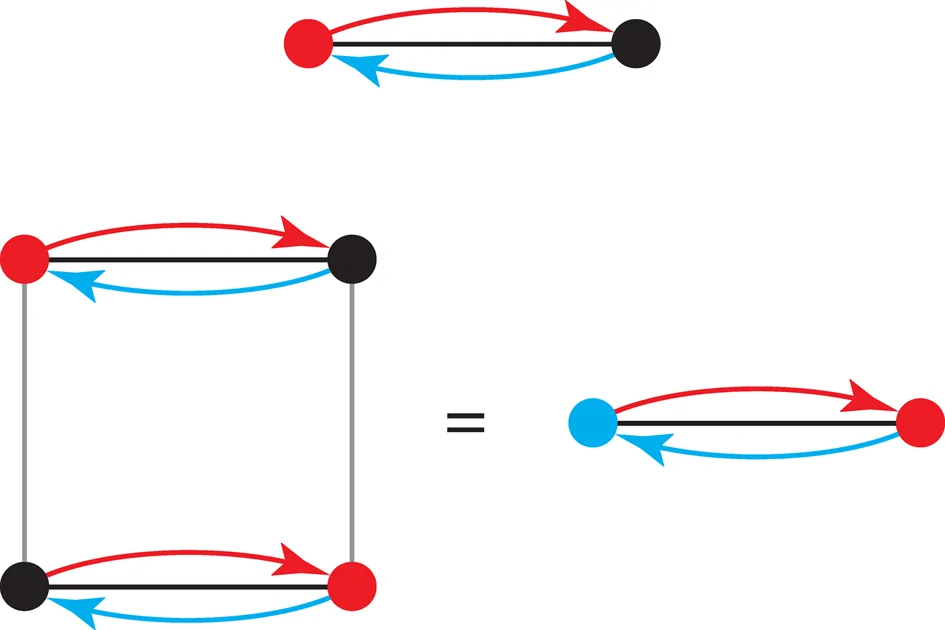
(Top) Labelled quotient graph of the net **hcb**, (bottom left) labelled quotient graph of the double net including the interaction between two opposite sheets represented as grey edges; oriented edges with voltage 10 (blue) and 01 (red). Red vertices define a maximum independent set. (Bottom right) A simpler representation with a different colour associated to independent subsets of each sheet.

**Figure 4:**
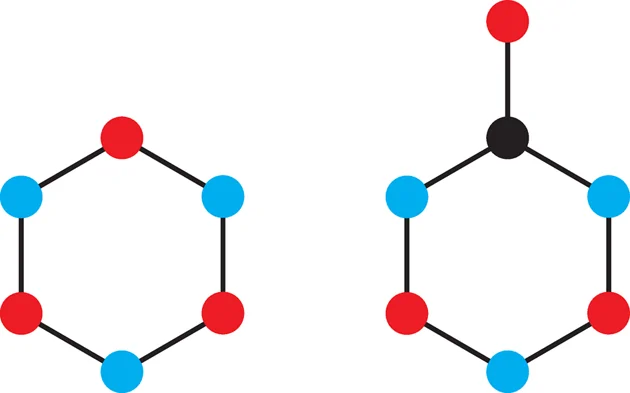
Ring configurations, from left to right: (0,0) and (1,0).

**Figure 5:**
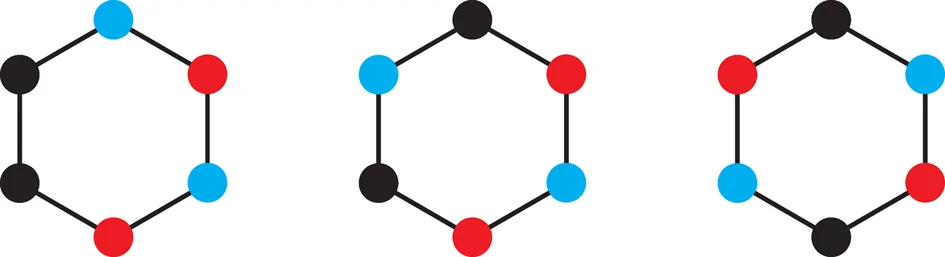
Ring configurations, from left to right: (2,1), meta-(2,0) and para-(2,0).

**Figure 6:**
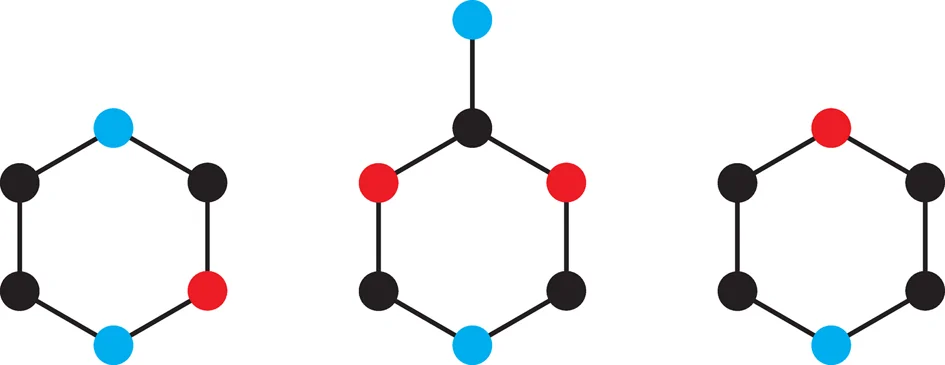
Ring configurations, from left to right: (3,1), (3,0) and (4,2).

**Figure 7:**
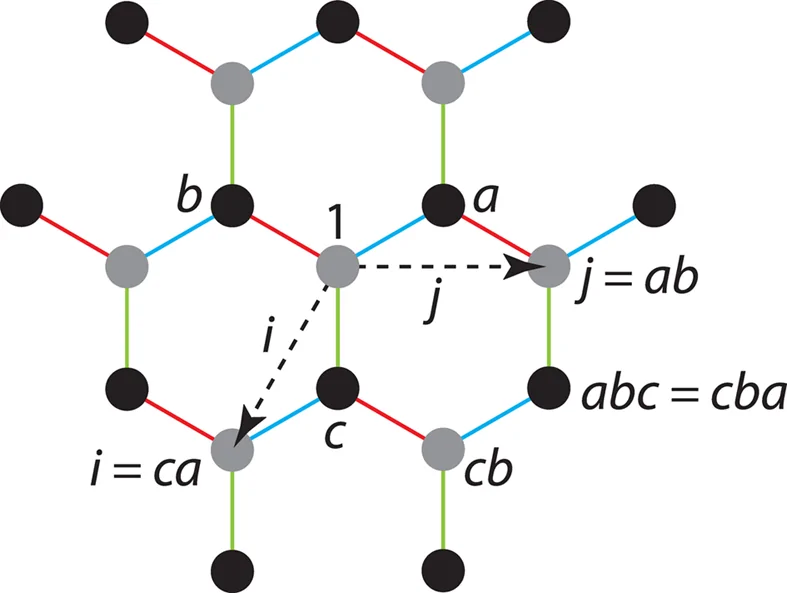
The **hcb** net as a Cayley colour graph; the three generators are represented as coloured edges: blue (*a*), red (*b*) and green (*c*). Vertex colours segregate the two parts of the bipartition. Basis vectors of the unit cell are defined as 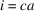 (10), 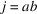 (01). Vertex labels can be assigned once the origin (1) has been fixed.

**Figure 8:**
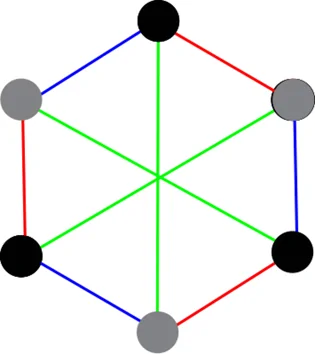
A Cayley colour graph of a quotient graph of **hcb** by a translation subgroup of index 3.

**Figure 9:**
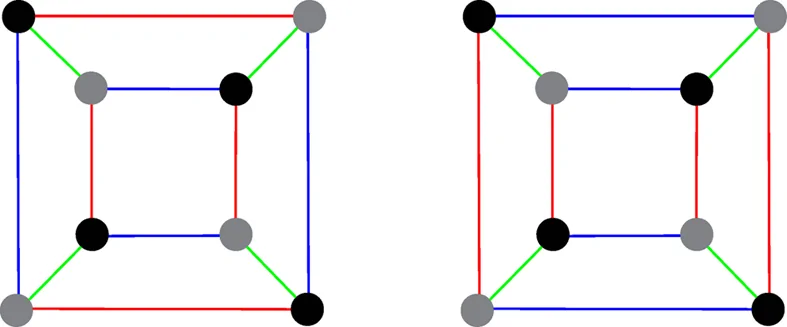
Cayley colour graphs of the two possible quotient graphs of **hcb** by a translation subgroup of index 4: (left) 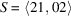 with two 2-colour 8-cycles and (right) 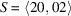 with only 2-colour 4-cycles.

**Figure 10:**
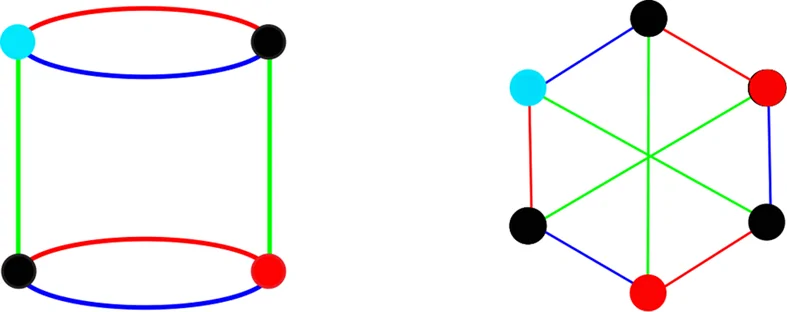
Two unique 2-colourings, up to isomorphism, of quotient graphs of **hcb** by subgroups of index 2 (left) 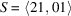 and 3 (right) 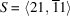.

**Figure 11:**
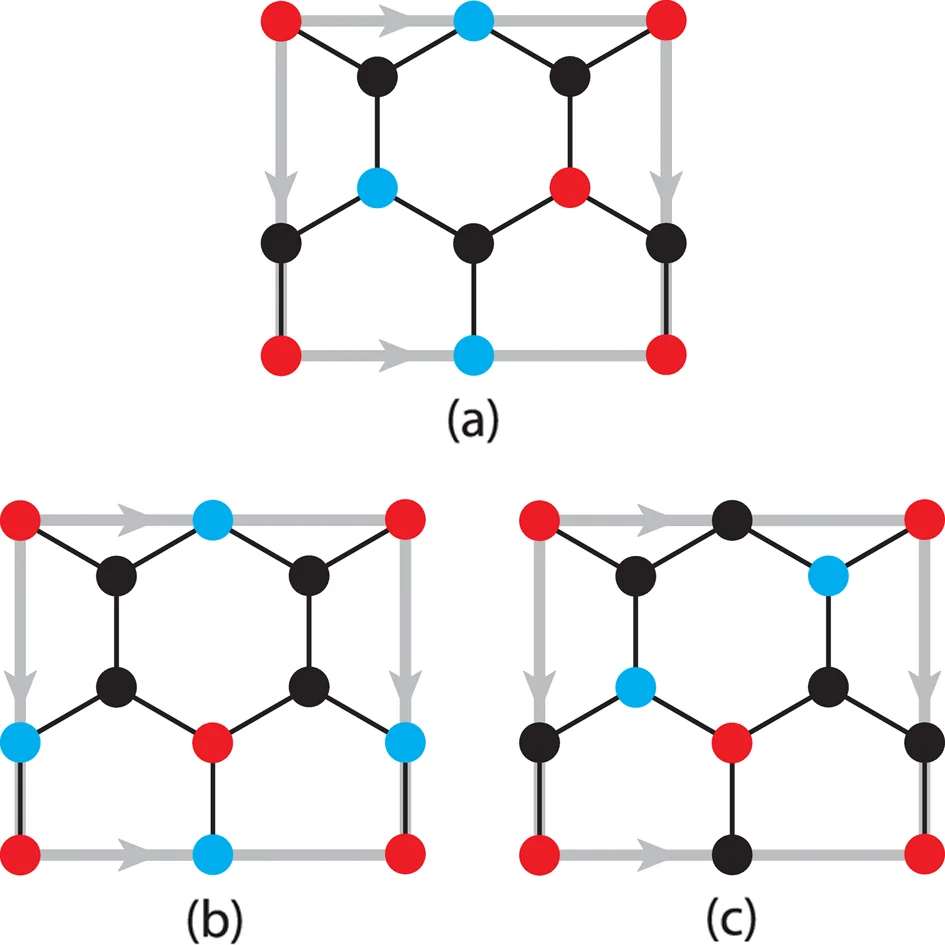
The three non-isomorphic configurations in the rectangular unit cell of **hcb** with *Z* = 4: (*a*) R06, (*b*) R03 and (*c*) R15.

**Figure 12:**
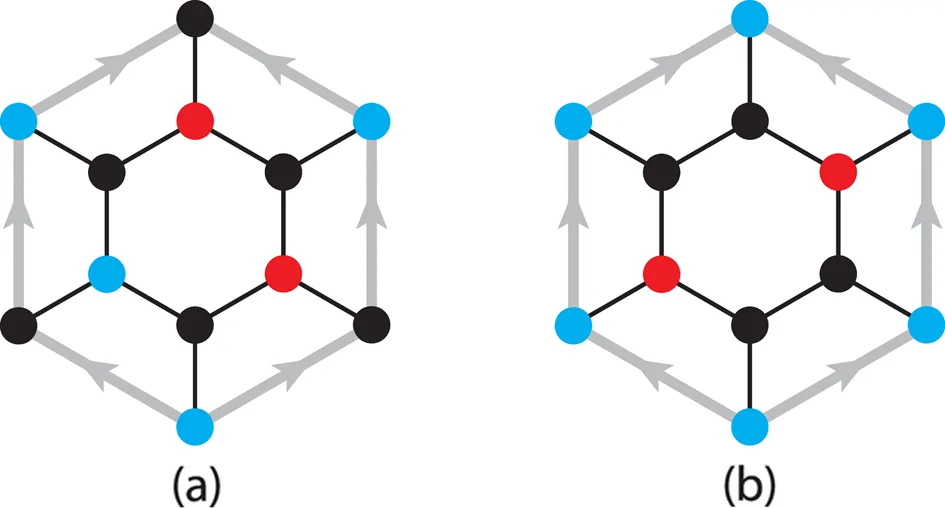
The two non-isomorphic configurations in the hexagonal unit cell of **hcb** with *Z* = 4: (*a*) H03 and (*b*) H09.

**Figure 13:**
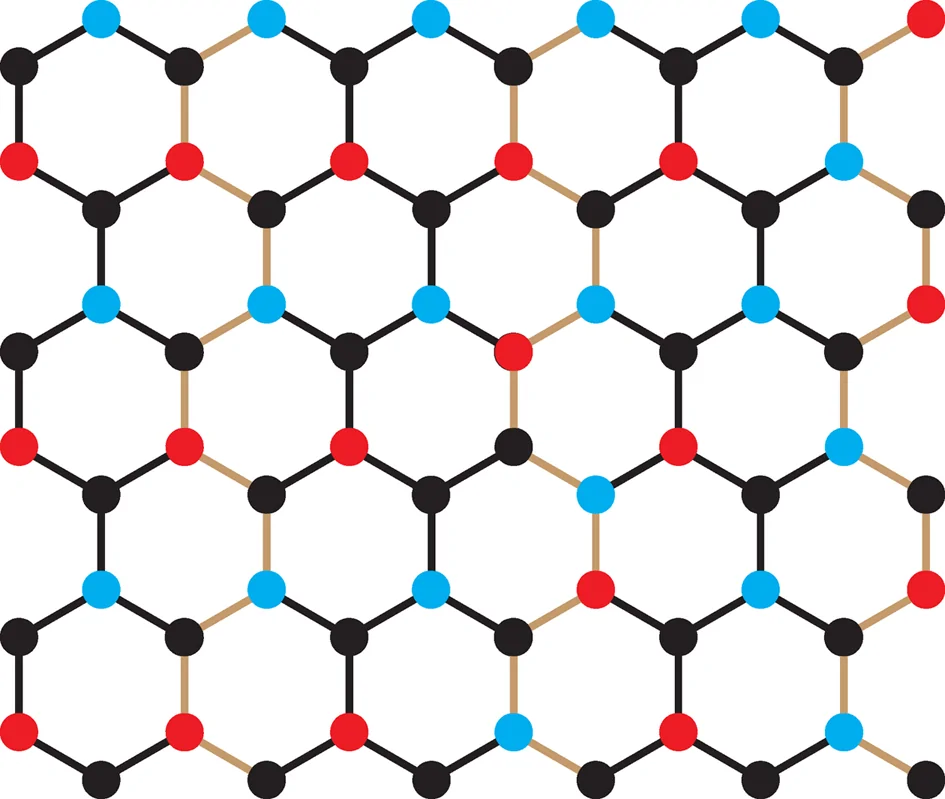
A fragment of a disordered **hcb** double layer designed from a single hyperedge with alveoli marked in brown.

**Figure 14:**
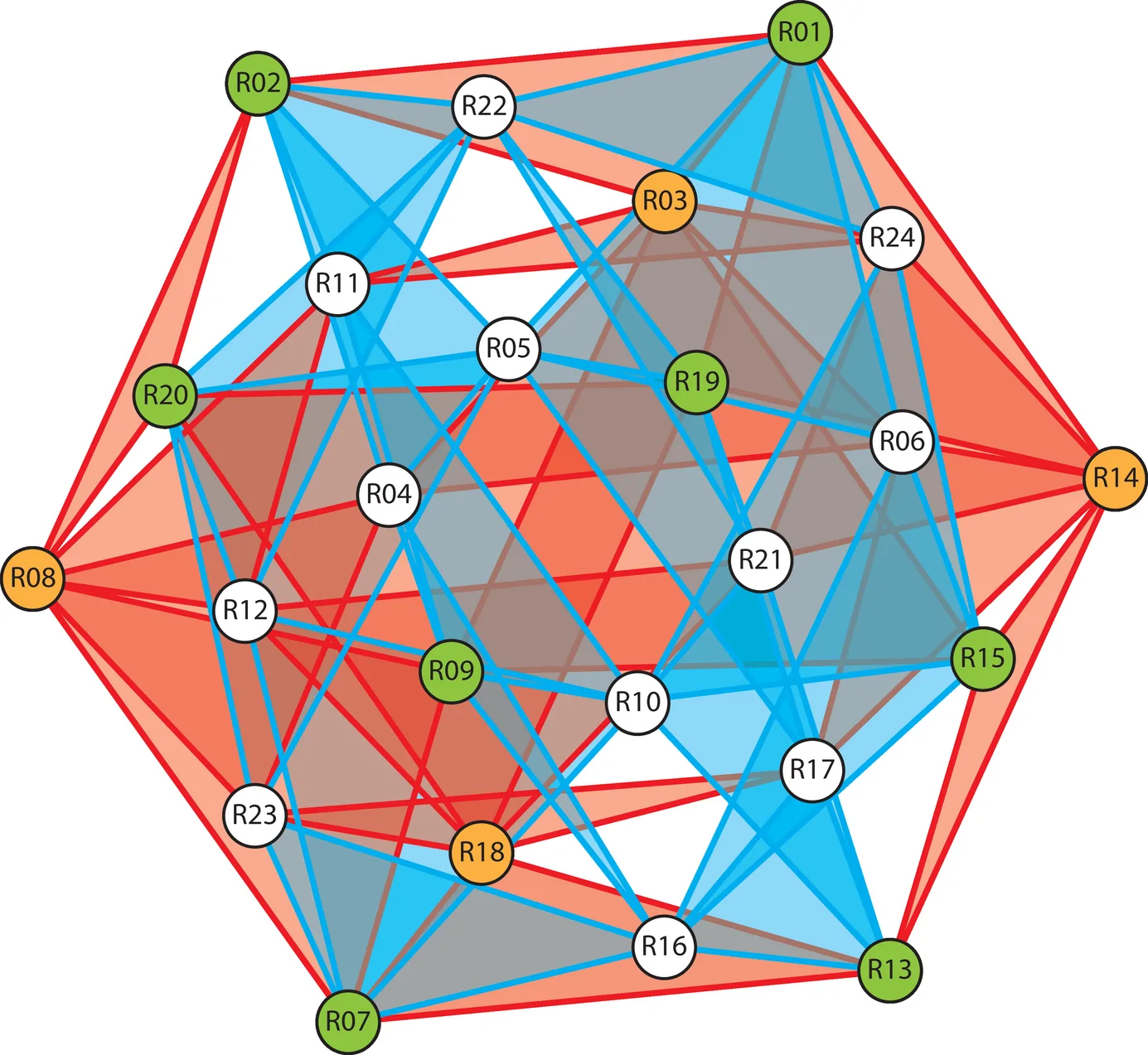
The hypergraph of correlations corresponding to the rectangular unit cell of the double **hcb** layer with *Z* = 4. In blue and red, hyperedges relative, respectively, to the 10 and 01 directions; in white, orange and green, vertices representing configurations equivalent to Figs. 11 ▸(*a*), 11 ▸(*b*) and 11 ▸(*c*), respectively.

**Figure 15:**
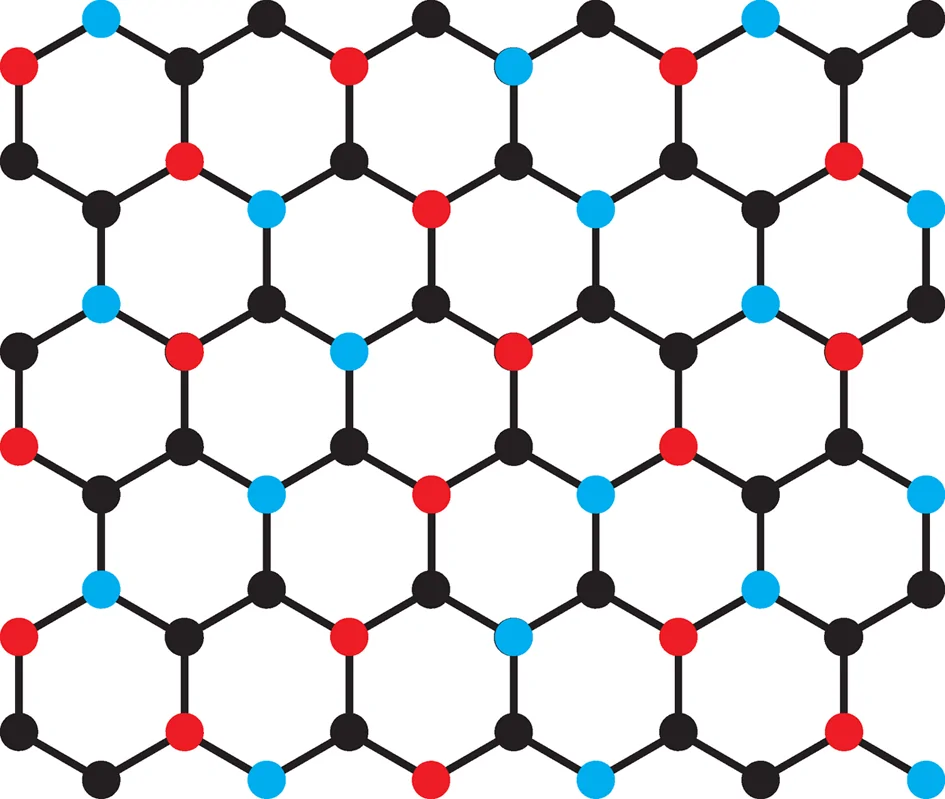
A fragment of a disordered **hcb** double layer designed from configurations R01, R02, R11 and R24.

**Figure 16:**
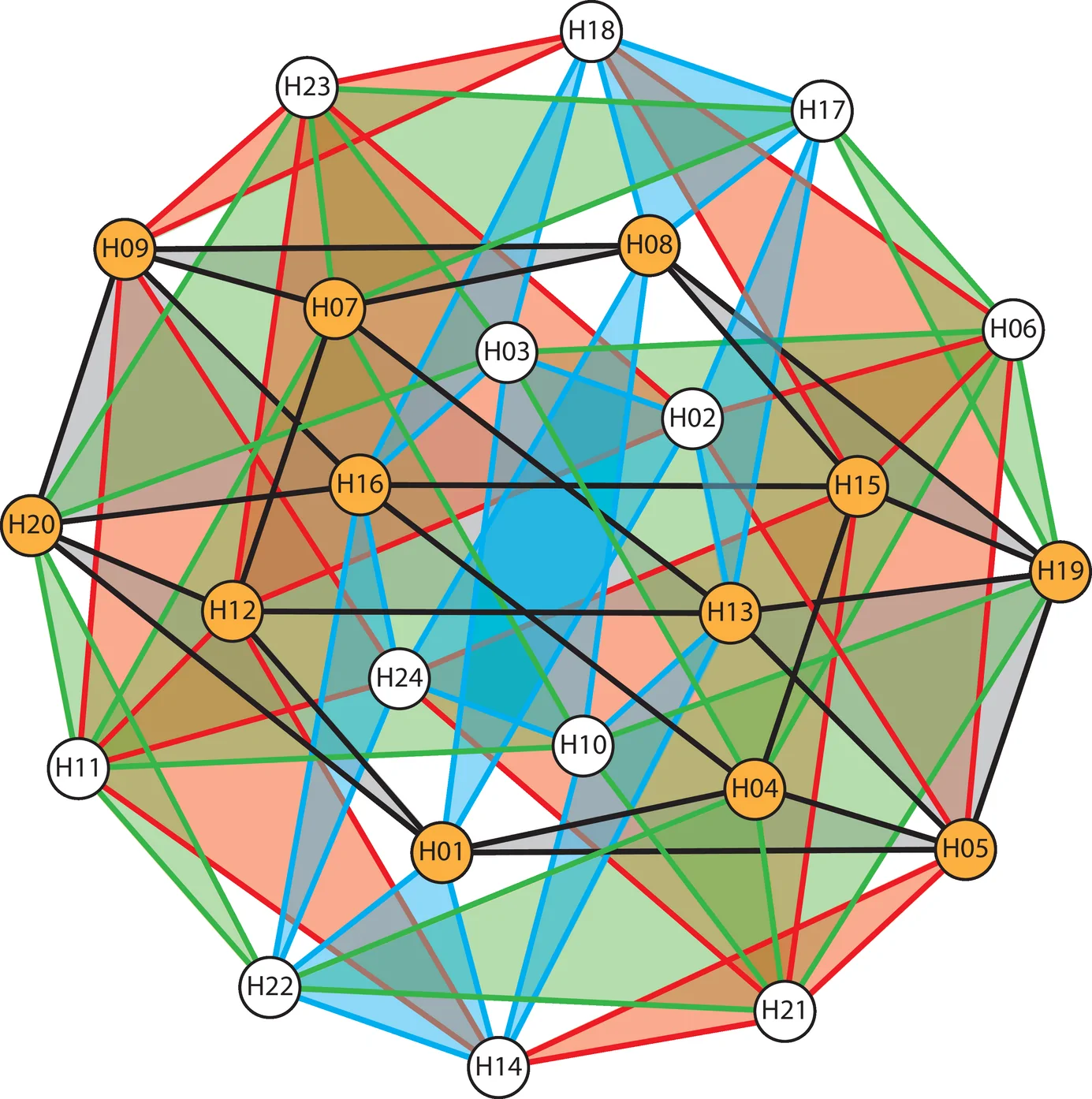
The hypergraph of correlations corresponding to the hexagonal unit cell of the double **hcb** layer with *Z* = 4. In blue, red and green, hyperedges relative, respectively, to the 10, 01 and 11 directions; in grey hyperedges relative to finite, hexagonal alveoli. In white and orange, vertices representing configurations equivalent to H24 and H20 shown, respectively, in Figs. 12 ▸(*a*) and 12 ▸(*b*).

**Figure 17:**
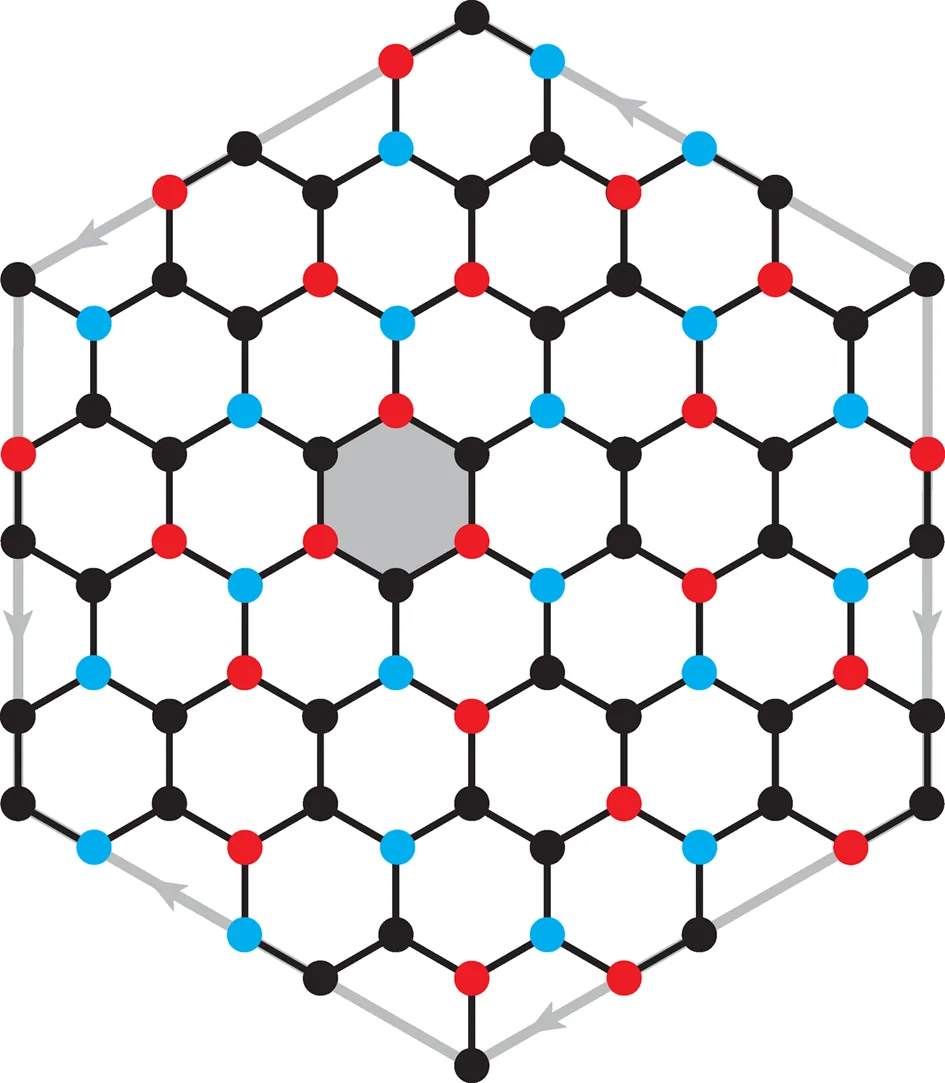
A fragment of a disordered **hcb** double layer designed from configurations H09, H16 and H20; the marked face with three red vertices creates non-stoichiometry.

**Figure 18:**
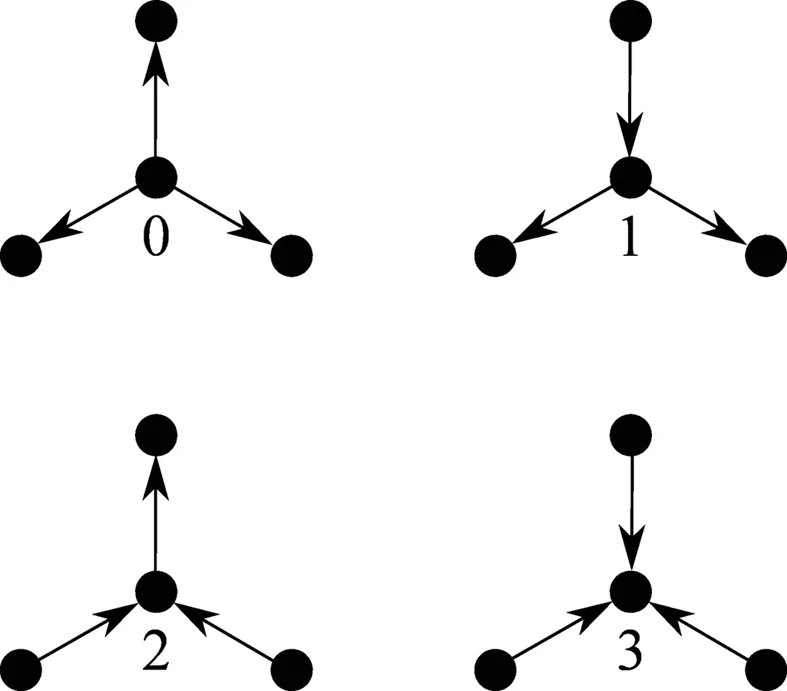
Resulting charge on the central vertex as a function of the orientations of incident edges.

**Figure 19:**
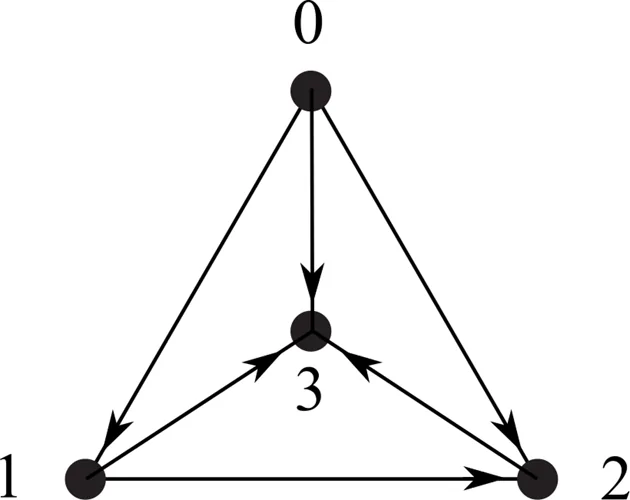
A digraph isomorphic to T_4_, to which maximal independent sets should be homomorphic, with vertices labelled by the respective charges.

**Figure 20:**
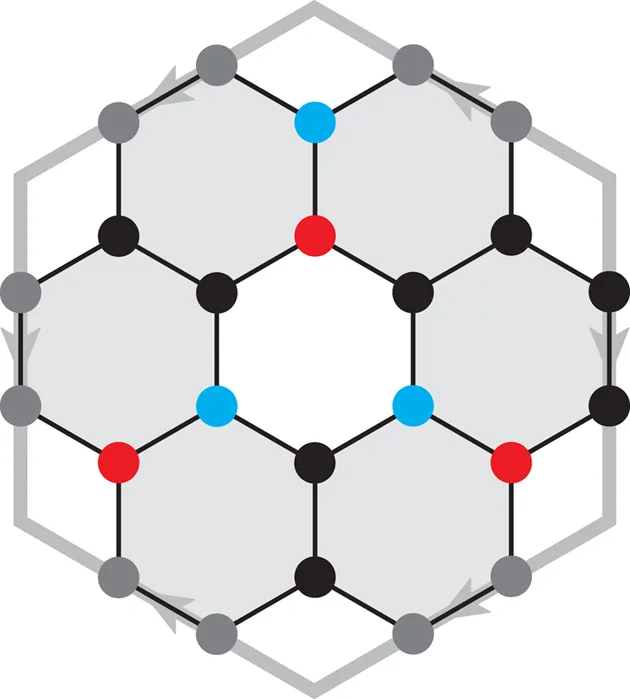
Toroidal representation of the quotient graph **hcb**/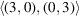 showing a forbidden Al/Si substitution with rings of type (3, 1) structured into a 6-cycle.

**Figure 21:**
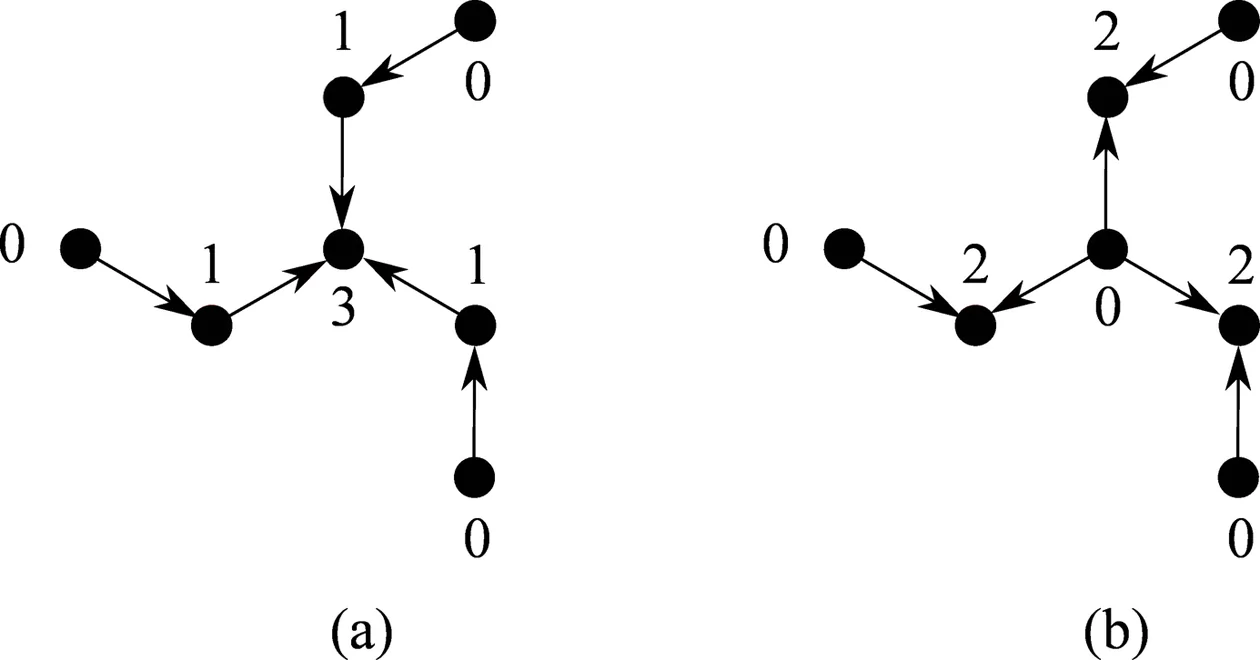
Two coloured subgraphs that can be extended to a homomorphism of the quotient graph **hcb**/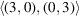 to T_4_.

**Table 1:** Comparison of observed (obs.) and theoretical (theor.) intensities of three components of the 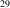
Si NMR spectra of synthetic phyllosilicates as expected from a single Al/Si sheet

Al/(Al+Si)	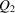 , obs. (theor.)	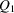 , obs. (theor.)	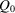 , obs. (theor.)
0.12	0 (0)	39 (41)	61 (59)
0.15	4 (0)	44 (53)	52 (47)
0.20	10 (0)	55 (75)	35 (25)
0.26	24 (0)	57 (100)	19 (0)

**Table 2:** Linear combinations of terms 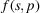 involved in the determination of the ID number of the quotient graph of the double **hcb** layer The coefficient of each term appears as the entry in the respective row. For instance, the entries of the first row 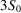 are given as 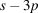 and those of the fourth row 3*S* are given as *s* according to equations (4) and (3), respectively.

	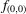	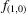	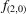	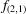	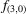	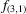	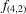
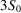	0	1	2	−1	3	0	−2
*C*	0	−2	2	−1	0	0	−2
3*F*	3	3	3	3	3	3	3
3*S*	0	1	2	2	3	3	4
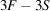	3	2	1	1	0	0	−1

## Appendix A Maximal independent sets of the double layer as T_4_-colourings of **hcb**

We want to characterize maximal independent sets in quotient graphs of the double layer of the **hcb** net. To this end, we look at the formation of the independence polynomial, a multivariate polynomial describing the full list of maximal independent sets of a graph, introduced by Moreira de Oliveira Jr & Eon (2021 ▸). We briefly recall the method, based on propositional calculus. To every vertex  of a graph, we associate an indeterminate , representing the proposition  (read *not* V) and meaning that site  is not occupied by an Al atom. The Löwenstein rule applied to the edge  is equivalent to proposition  and  =  or , which is written in the polynomial form . Usual operations on polynomials substitute logical operations:  (*or*) for addition and  (*and*) for multiplication. The independence polynomial *P* of the graph is defined as the product  over the set of edges. Every monomial in the development of *P* describes an independent set. Of course, powers of the indeterminate  can be substituted by the linear term since  (*and*) is the same as (). Moreover, two monomials of the form  and  are, respectively, associated to a set and one of its subsets. Looking for maximal independent sets, we may delete the latter. This simplification can be performed after or during the development of the polynomial.

In the double **hcb** layer, every edge  in one layer is duplicated by an edge  in the other layer, with two links  and  between the two layers. Associating the indeterminates  and  to vertices  and , we form the partial polynomial:

which gives, after development and simplification,

The product over the whole edge set of **hcb** provides the list of independent sets of the double layer. (Notice that setting  for all *i* yields the independence polynomial of the single layer.) It can be seen that each edge of **hcb** contributes with one of the two terms  or  to this polynomial. We can thus define a pictorial representation of any independent set of the double layer by choosing an orientation for every edge  with tail marked as *x* and head as *y*. The terms  and  are, respectively, represented as an arc from  to  and an arc from  to  in the net **hcb**. Conversely, any orientation of **hcb** determines an independent set of the double layer.

Consider then, in **hcb**, a vertex  and its three neighbours ,  and . Depending on the local orientation of the three incident edges, the neighbourhood of , associated to the product , contributes to the independence polynomial with eight terms equivalent to one of the four products  (a single term),  (three terms),  (three terms) or  (a single term). The first term with all arcs outgoing from  forbids the presence of Al on the *x*, say the front layer, and on its neighbours on the back (*y*) layer, hence the vertex on the back layer is occupied by Al in a maximal independent set associated with such a term. Similarly, the fourth term corresponds to Al on the front layer. The second and third terms forbid the presence of Al in both layers: both sites, at the front and at the back, are occupied by Si.

A simple interpretation is afforded by the *charging method*. Initially, given any orientation of the graph, we give every edge charge 1. Then each edge is discharged to one of its end-vertices, according to the orientation of the respective edge, as schematized in Fig. 18 ▸. The resulting charge on a vertex varies from 0 to 3: according to the previous discussion, vertices with charge 0 and 3 correspond to Al atoms on the back and front layer, respectively, with Si on the opposite layer. Vertices with charge 1 or 2 are Si atoms on both layers. The method ensures that the respective Al/Si distribution is independent; clearly adjacent vertices cannot both receive the same charge, 0 or 3.

As seen at the end of Section 5, an independent set of the double layer is maximal whenever any Si vertex, *i.e.* any vertex of charge 1 or 2 in the pictorial **hcb** representation, has at least one Al neighbour in each layer, *i.e.* one of charge 0 and one of charge 3 in the **hcb** representation. It is easily verified that this condition (the *condition of maximality*) prevents the existence of (i) edges between vertices with the same charge 1 or 2 and (ii) edges oriented from a vertex of charge 2 to a vertex of charge 1. This amounts to saying that an orientation of some quotient graph of the **hcb** net satisfying the condition of maximality describes a maximal independent set of the double layer if and only if there is a graph homomorphism from the respective digraph to the digraph T_4_ (a *tournament* graph) shown in Fig. 19 ▸. We then say that the graph admits a T_4_-colouring. Let us call source and sink the vertices of charge 0 and 3, respectively. We may sum up these considerations as follows.

Proposition A.1 An orientation in a quotient graph of **hcb** describes a maximal independent set of the double **hcb** layer if and only if (i) the respective digraph is homomorphic to T_4_ and (ii) any vertex that is neither a source nor a sink is linked to at least one source and one sink.

We now draw several consequences of Proposition A.1[Statement propositiona.1]. But first, we note that the set of edges of the quotient graph of a given colour forms a matching, and because the ID number of the double **hcb** layer corresponds to the ratio Al/Si = 1 of the **hcb** layer, it follows that the number of Si–Si links along any direction (colour) of the edges of the quotient graph must be equal to the number of Al–Al links along the same direction (colour).

Let us call a *leg* of a Si–Si link any of the four associated Si–Al links.

Proposition A.2 In a T_4_-colouring of a quotient graph of **hcb** the length of a cycle formed by the legs of some Si–Si links is a multiple of 4.

Proof By homomorphism, this cycle is mapped to a closed walk running along the 4-cycle of T_4_ obtained after deleting the arcs  and . This walk follows one of the two directed paths of length 2, from vertex 0 to vertex 3 along the legs, forward then backward but not necessarily along the same path, until it closes at vertex 0. □

Proposition A.3 The total number of Al–Al and Si–Si links per cycle of a quotient graph of **hcb** is even.

Proof Because cycles of the quotient graph are even they are mapped to even closed walks in T_4_. Let us call  the number of times the edge *ij* is crossed, in one direction or the other, in such a closed walk: we have  in the field . Moreover, every time a walk crosses some vertex, it runs along an ingoing and an outgoing edge, hence the sum of  terms from the incident edges is also even. In particular  at vertex 3. Inserting this expression in the previous equation yields . Since we also have  at vertex 0, we can add the last two equations to get . □

Looking at the five acceptable ring configurations in Figs. 5 ▸ and 6 ▸, we can see that the total number of Al–Al and Si–Si links per ring is 0 or 2, in accordance with the previous result. This means that each ring containing some link is adjacent to two rings also containing two links. Since rings of the graph form the vertices of its dual, and every edge of the graph corresponds to an edge of its dual, we deduce that those rings with links are associated to cycles of the dual.

Otherwise, it can be useful to define some specific digraph as a subgraph we want to find in the quotient graph *G* of **hcb** when this digraph characterizes a family of maximal independent sets. We may, for example, analyse digraphs associated to subgraphs induced by a subset of vertices of given charges. Calling *D* a digraph representing some local feature of a maximal independent set, we will be looking for the existence of injective homomorphisms  that can be extended to a homomorphism . These properties can help determine the nature and number of maximal independent sets in quotient graphs of small order. Two complete examples are dealt with in the next sections[App appb][App appc].

## Appendix B **hcb**/〈(2,0),(0,2)〉

We consider the quotient graph shown in Fig. 9 ▸, which is also that of **hcb**/, with eight vertices and 12 edges. For a given homomorphism  denote  the vertex set mapped to vertex *i* in T_4_ for  and call ,  and  the cardinalities of these sets. Maximal independent sets corresponding to Al/Si = 1 satisfy the following two equations, where the second one is a balance of charges:

This system admits a single solution noted as the pair . We look at a solution with a Si–Si link; according to Proposition A.3 there must be a parallel Al–Al link in each of the two 4-cycles sharing the respective edge. Moreover, one of these two Al–Al links is shared by a third 4-cycle which must then also contain one Si–Si link. This observation discards the possibility of a solution with a single Si–Si link. It is easily seen that there are 12 2-colourings of the quotient graph with parallel pairs of Al–Al links mapped to the arc  of T_4_. In solutions with no Al–Al links Al occupies a whole part of the bipartition of the quotient graph giving again 12 2-colourings with . The 24 2-colourings are drawn in the supporting information.

## Appendix C **hcb**/〈(3,0),(0,3)〉

Fig. 20 ▸ shows the quotient graph , with 18 vertices and 27 edges which should satisfy the following equations:

This system admits four solutions given by the pair : (0, 6) and (9, 3) are equivalent through inversion of all orientations in *G* and similarly are (6, 4) and (3, 5).

We begin with , which cannot present Si–Si links (), and thus no Al–Al links either, that is: no edges of *G* are mapped to the arcs  and . Hence, the nine vertices in  correspond to a whole part of the bipartition of *G*. Let us consider the neighbourhood of some vertex in , as shown in Fig. 21 ▸(*a*). The first neighbours, which are in , each have a single neighbour in . Note that any two vertices from  must be at distance 4, otherwise the separating vertex would belong to . Fig. 21 ▸(*a*) shows the only solution, up to isomorphism, with the three vertices from  at mutual distance 4. An analysis of the quotient graph reveals that there are indeed six such 3-vertex subsets, each providing a homomorphism to T_4_ by extension.

Let us consider now the second case . There can exist as many as three Si–Si links () and three Al–Al links forming up to two 3-cycles, one based on rings of type (4, 2) and the other on rings of type para-(2, 0), or one 6-cycle based on rings of type (3, 1). According to Proposition A.2 both solutions are forbidden. The latter case is illustrated in Fig. 20 ▸.

To determine solutions without Si–Si links, we look at all possible subgraphs induced by vertices from  (four vertices) and  (three vertices) or, more exactly, possible subgraphs induced by the arcs . Such subgraphs include 6-cycles and 7-paths that cannot be extended to a homomorphism to T_4_. Only the branched tree shown in Fig. 21 ▸(*b*) is easily seen to extend to a homomorphism. Indeed, remember that we just need to add the six vertices from  given as end-vertices of outgoing arcs  and closing with vertices from  given as end-vertices of outgoing arcs  and , always following the underlying **hcb** net. This algorithm will generate more than five vertices of charge 3 but it can be verified that only five are translationally non-equivalent. Two such isomorphic branched trees can be centred at each of the 18 vertices of the quotient, yielding hence 36 possible configurations. Summing up, we find 84 configurations with Al/Si = 1.
