# Supplementary material for: Maximal independence and regularity of Al/Si distributions in disordered phyllosilicates 2:1
Source: Acta Crystallogr A Found Adv. 2026 Oct 15;82(Pt 6):472–85. doi: 10.1107/S2053273326009794 (PMC13720708; doi:10.1107/S2053273326009794)
Supplement: Supplementary file 1 [file a-82-00472-sup1.pdf]

Acta Cryst A FOUNDATIONS ADVANCES

Volume 82 (2026)

Supporting information for article:

Maximal independence and regularity of Al/Si distributions in disordered phyllosilicates 2:1

Montauban Moreira de Oliveira Jr and Jean-Guillaume Eon

Generating the correlation hypergraphs

This material explains the construction of the correlation hypergraphs associated with the two labeled quotient graphs of order 8 of **hcb**. We describe the generation of the alveoli, a tool developed to model Si/Al distributions in accordance with the principle of maximal independence. Theoretical justifications can be found in (Moreira de Oliveira Jr & Eon, 2025).

We call *i*) *configuration* a maximal independent set of vertices in a given graph and *ii*) *family of configurations*, a subset of configurations with the same cardinality. Hypergraphs are mathematical structures analogous to graphs, possessing vertices and hyperedges. While in a graph each edge links two vertices, each hyperedge in a hypergraph may cover more than two vertices. As vertices of the correlation hypergraph, we take the configurations of a given family of configurations F. A hyperedge h is then defined as a subset of configurations $F_h \subset F$ with maximal overlap O_h. More exactly, the hyperedge h is given as the set F_h of configurations such that $C_i \cap C_j = O_h$ for any pair C_i, C_j in F_h, where O_h has maximal cardinality in the quotient graph.

In order to study disordered Al/Si distributions in $2:1$ phyllosilicates with Si/Al=3, we apply this procedure to the quotient graph G of the **hcb** net considering the rectangular unit cell shown in Figure 1.

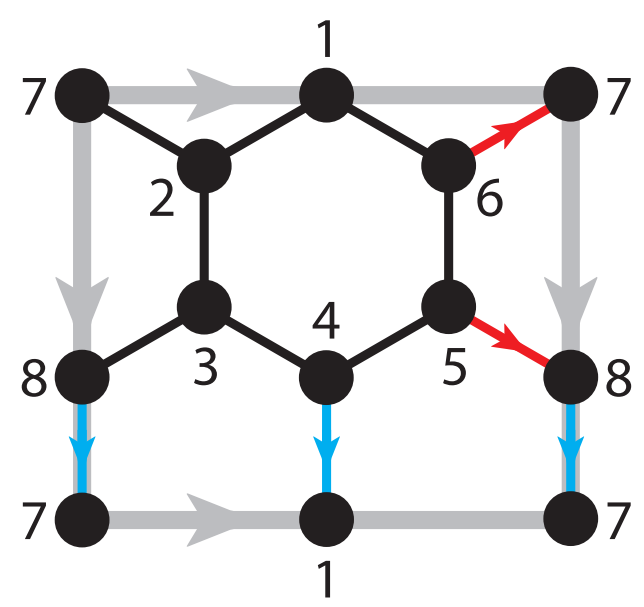

Figure 1: Labelled quotient graph corresponding to the rectangular unit cell of **hcb** in relation to a translation subgroup of index 4; blue and red edges correspond respectively to vector labels 10 and 01.

This graph admits 42 configurations: 24 with cardinality 4, 16 with cardinality 5, and 2 with cardinality 8. As we want to investigate the case Si/Al=3, we consider exclusively the 24 configurations with cardinality 4 that are described in Figure 2. The vertices in a configuration are denoted

by their label in the quotient graph of **hcb**, followed by the letter (B) or (R), according to whether it is colored blue or red, respectively.

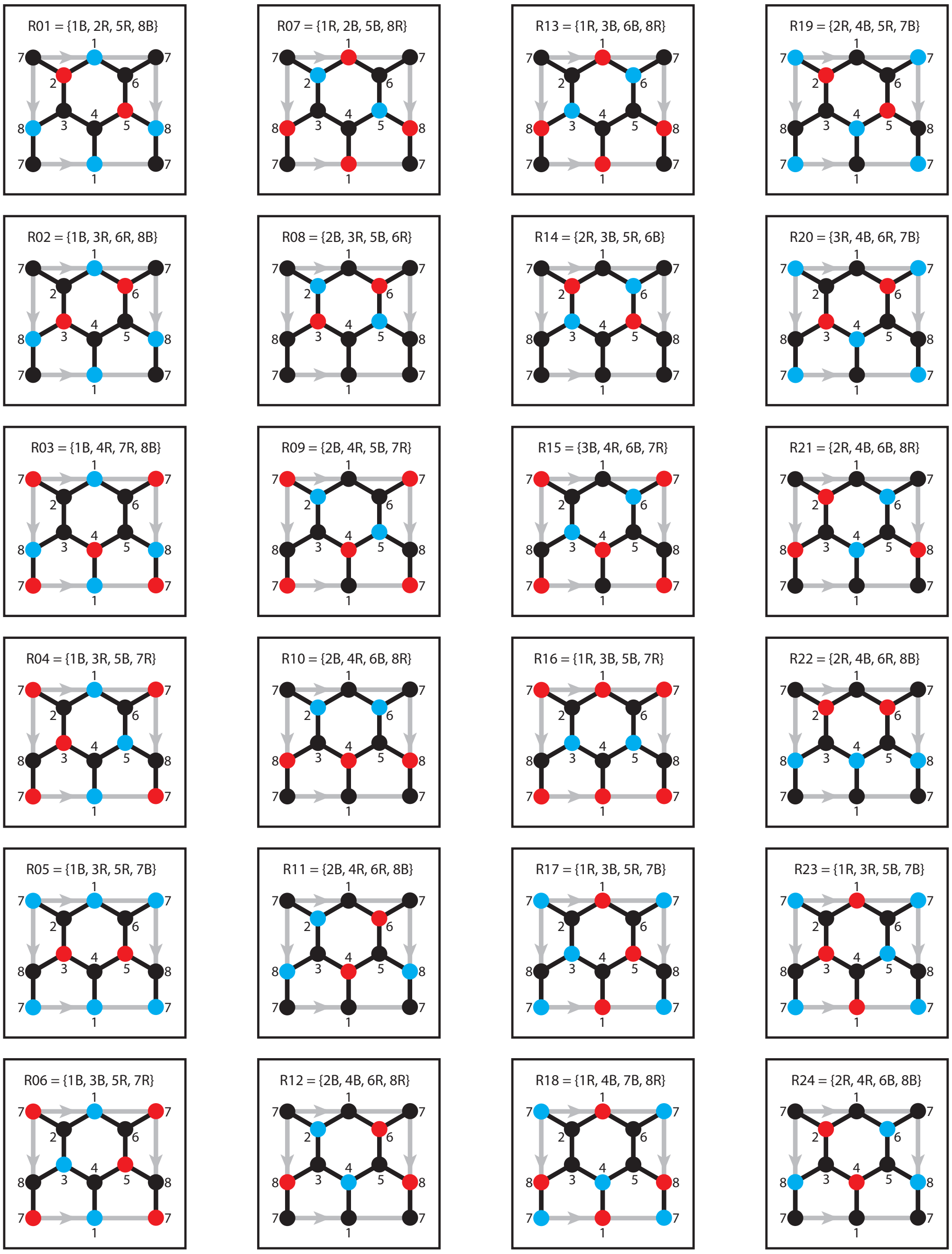

Figure 2: List of configurations of cardinality 4 corresponding to the rectangular unit cell.

The maximum intersection between any pair of configurations has cardinality 2, and there are 32 distinct sets of three configurations that reach this maximum overlap value. Sets with common intersection define the hyperedges.

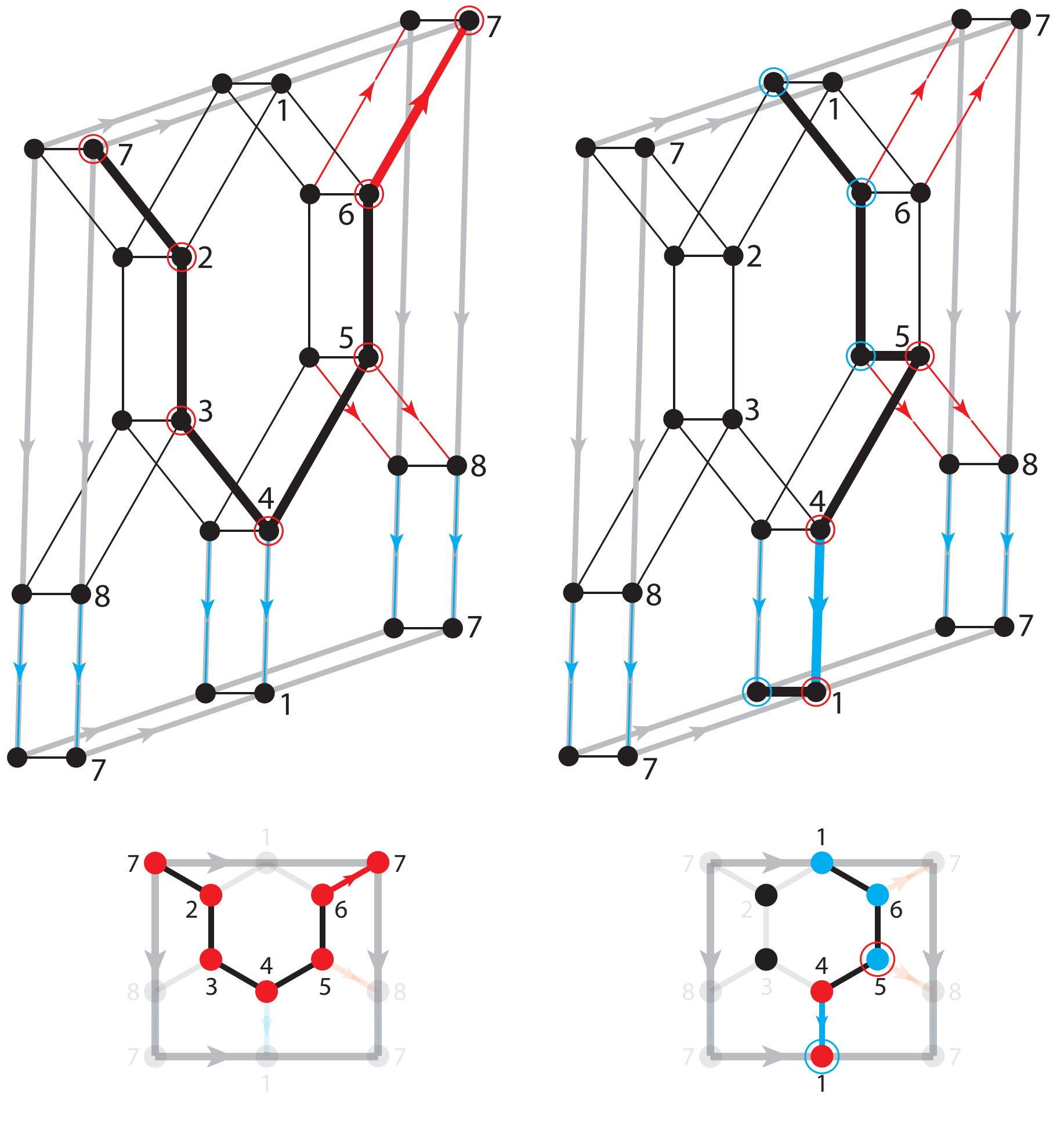

Figure 3: The shaded subgraphs represent the cycle 2R-3R-4R-5R-6R-7R with net voltage 01 in (a) and the cycle 1B-6B-5B-5R-4R-1R with net voltage 10 in (b).

Given the intersection $O = C_1 \cap C_2 \cap C_3$ of three configurations covered by a hyperedge, we call $I_G(O)$ the subgraph induced by the vertices belonging to the complement in the quotient graph of $O \cup N(O)$, the union of O and its neighbours $N(O)$. Because pairwise intersections are all equal, we

may also define $I_G(O)$ as the subgraph induced by the symmetric difference $(C_1 \cup C_2 \cup C_3) - O$. The lift of $I_G(O)$ in the **hcb** net is generally a disconnected graph, each component of which is called an alveolus. Alveoli may be finite, or periodic subgraphs, according to the translation subgroup generated by the net voltages over the cycles in $I_G(O)$.

As an example, we examine the hyperedge $\{R01, R02, R03\}$ with overlap $O = \{1B, 8B\}$ and symmetric difference $\{2R, 3R, 4R, 5R, 6R, 7R\}$, inducing a 6-cycle C_6 with net voltage 01, as seen in Figure 2. For a better visualization, Figure 3 shows the quotient graph of the double **hcb** net, distinguishing the two layers. Note that the cycle C_6 is entirely in the same layer (Figure 3(a)). As another example, we examine the hyperedge $\{R15, R16, R06\}$. In this case, $I_G(O)$ is the cycle $C_6 = 1B - 6B - 5B - 5R - 4R - 1R$, represented in Figure 3(b), with net voltage 10. In both cases the alveolus is a 1-periodic line along the respective voltage.

We list below all hyperedges, 16 with voltage 10 and 16 with voltage 01.

The respective correlation hypergraph can be seen in Figure 4, with triangles representing the hyperedges.

The same procedure can be repeated with the quotient graph of the **hcb** net in relation to the hexagonal unit cell, shown in Figure 5, and leading to the 24 configurations with cardinality 4 described in Figure 6.

Again 32 sets of three configurations reach the maximum overlap of 2 vertices and define the hyperedges as listed below. The associated configuration hypergraph can be seen in Figure 7, with triangles representing the hyperedges. It is worth observing that finite alveoli are present in this case together with 1-periodic alveoli running along directions 10, 01 and 11.

Table 1: Hyperedges with associated sets O, induced graph $I_G(O)$, and voltage.

Hyperedge	O	$I_G(O)$	Voltage
$\{R01, R02, R03\}$	$\{1B, 8B\}$	C_6	01
$\{R06, R04, R03\}$	$\{1B, 7R\}$	C_6	01
$\{R07, R08, R09\}$	$\{2B, 5B\}$	C_6	01
$\{R12, R08, R11\}$	$\{2B, 6R\}$	C_6	01
$\{R13, R14, R15\}$	$\{3B, 6B\}$	C_6	01
$\{R06, R14, R17\}$	$\{3B, 5R\}$	C_6	01
$\{R18, R19, R20\}$	$\{4B, 7B\}$	C_6	01
$\{R04, R08, R23\}$	$\{5B, 3R\}$	C_6	01
$\{R12, R21, R18\}$	$\{4B, 8R\}$	C_6	01
$\{R14, R21, R24\}$	$\{6B, 2R\}$	C_6	01
$\{R17, R18, R23\}$	$\{7B, 1R\}$	C_6	01
$\{R03, R11, R24\}$	$\{8B, 4R\}$	C_6	01
$\{R07, R13, R18\}$	$\{1R, 8R\}$	C_6	01
$\{R01, R14, R19\}$	$\{2R, 5R\}$	C_6	01
$\{R02, R08, R20\}$	$\{3R, 6R\}$	C_6	01
$\{R03, R09, R15\}$	$\{4R, 7R\}$	C_6	01
$\{R04, R05, R02\}$	$\{1B, 3R\}$	C_6	10
$\{R06, R05, R01\}$	$\{1B, 5R\}$	C_6	10
$\{R09, R10, R11\}$	$\{2B, 4R\}$	C_6	10
$\{R12, R07, R10\}$	$\{2B, 8R\}$	C_6	10
$\{R16, R13, R17\}$	$\{3B, 1R\}$	C_6	10
$\{R06, R16, R15\}$	$\{3B, 7R\}$	C_6	10
$\{R21, R19, R22\}$	$\{4B, 2R\}$	C_6	10
$\{R12, R20, R22\}$	$\{4B, 6R\}$	C_6	10
$\{R04, R09, R16\}$	$\{5B, 7R\}$	C_6	10
$\{R07, R16, R23\}$	$\{5B, 1R\}$	C_6	10
$\{R10, R15, R24\}$	$\{6B, 4R\}$	C_6	10
$\{R10, R13, R21\}$	$\{6B, 8R\}$	C_6	10
$\{R05, R20, R23\}$	$\{7B, 3R\}$	C_6	10
$\{R05, R17, R19\}$	$\{7B, 5R\}$	C_6	10
$\{R01, R22, R24\}$	$\{8B, 2R\}$	C_6	10
$\{R02, R11, R22\}$	$\{8B, 6R\}$	C_6	10

Table 2: Hyperedges with associated sets O, induced graph $I_G(O)$, and voltage.

Hyperedge	O	$I_G(O)$	Voltage
$\{H01, H02, H03\}$	$\{1B, 3R\}$	C_6	10
$\{H14, H13, H10\}$	$\{2B, 8R\}$	C_6	10
$\{H17, H08, H18\}$	$\{3B, 1R\}$	C_6	10
$\{H01, H14, H22\}$	$\{4B, 6R\}$	C_6	10
$\{H02, H13, H17\}$	$\{5B, 7R\}$	C_6	10
$\{H03, H18, H16\}$	$\{7B, 5R\}$	C_6	10
$\{H22, H24, H16\}$	$\{8B, 2R\}$	C_6	10
$\{H10, H08, H24\}$	$\{6B, 4R\}$	C_6	10
$\{H06, H05, H02\}$	$\{1B, 7R\}$	C_6	01
$\{H14, H12, H11\}$	$\{2B, 6R\}$	C_6	01
$\{H06, H15, H18\}$	$\{3B, 5R\}$	C_6	01
$\{H02, H12, H23\}$	$\{5B, 3R\}$	C_6	01
$\{H18, H23, H09\}$	$\{7B, 1R\}$	C_6	01
$\{H15, H21, H24\}$	$\{6B, 2R\}$	C_6	01
$\{H11, H24, H09\}$	$\{8B, 4R\}$	C_6	01
$\{H05, H14, H21\}$	$\{4B, 8R\}$	C_6	01
$\{H07, H10, H11\}$	$\{2B, 4R\}$	C_6	11
$\{H06, H04, H03\}$	$\{1B, 5R\}$	C_6	11
$\{H04, H21, H22\}$	$\{4B, 2R\}$	C_6	11
$\{H06, H17, H19\}$	$\{3B, 7R\}$	C_6	11
$\{H07, H17, H23\}$	$\{5B, 1R\}$	C_6	11
$\{H10, H19, H21\}$	$\{6B, 8R\}$	C_6	11
$\{H03, H23, H20\}$	$\{7B, 3R\}$	C_6	11
$\{H11, H22, H20\}$	$\{8B, 6R\}$	C_6	11
$\{H07, H08, H09\}$	$\{1R, 4R\}$	C_6	00
$\{H04, H01, H05\}$	$\{1B, 4B\}$	C_6	00
$\{H07, H12, H13\}$	$\{2B, 5B\}$	C_6	00
$\{H04, H15, H16\}$	$\{2R, 5R\}$	C_6	00
$\{H08, H15, H19\}$	$\{3B, 6B\}$	C_6	00
$\{H01, H12, H20\}$	$\{3R, 6R\}$	C_6	00
$\{H09, H16, H20\}$	$\{7B, 8B\}$	C_6	00
$\{H05, H13, H19\}$	$\{7R, 8R\}$	C_6	00

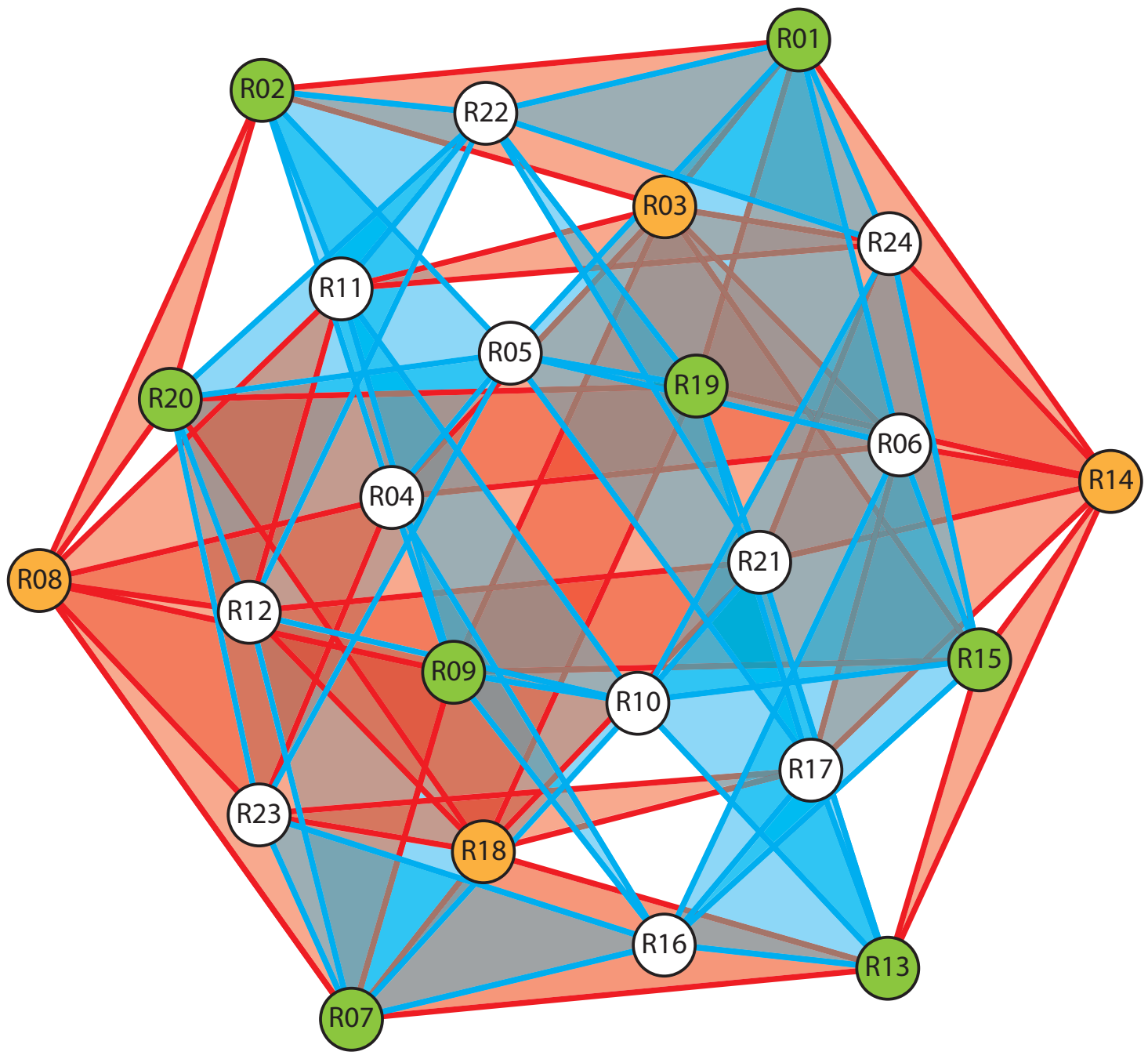

Figure 4: Correlation hypergraph corresponding to the rectangular unit cell. Blue and red triangles are hyperedges associated with alveoli in direction 10 and 01, respectively.

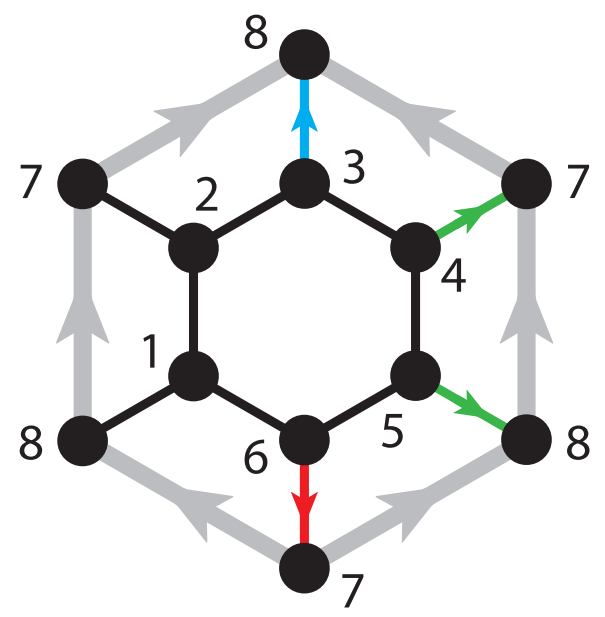

Figure 5: Labelled quotient graph corresponding to the hexagonal unit cell; blue, red and green edges correspond respectively to vector labels 10, 01 and 11.

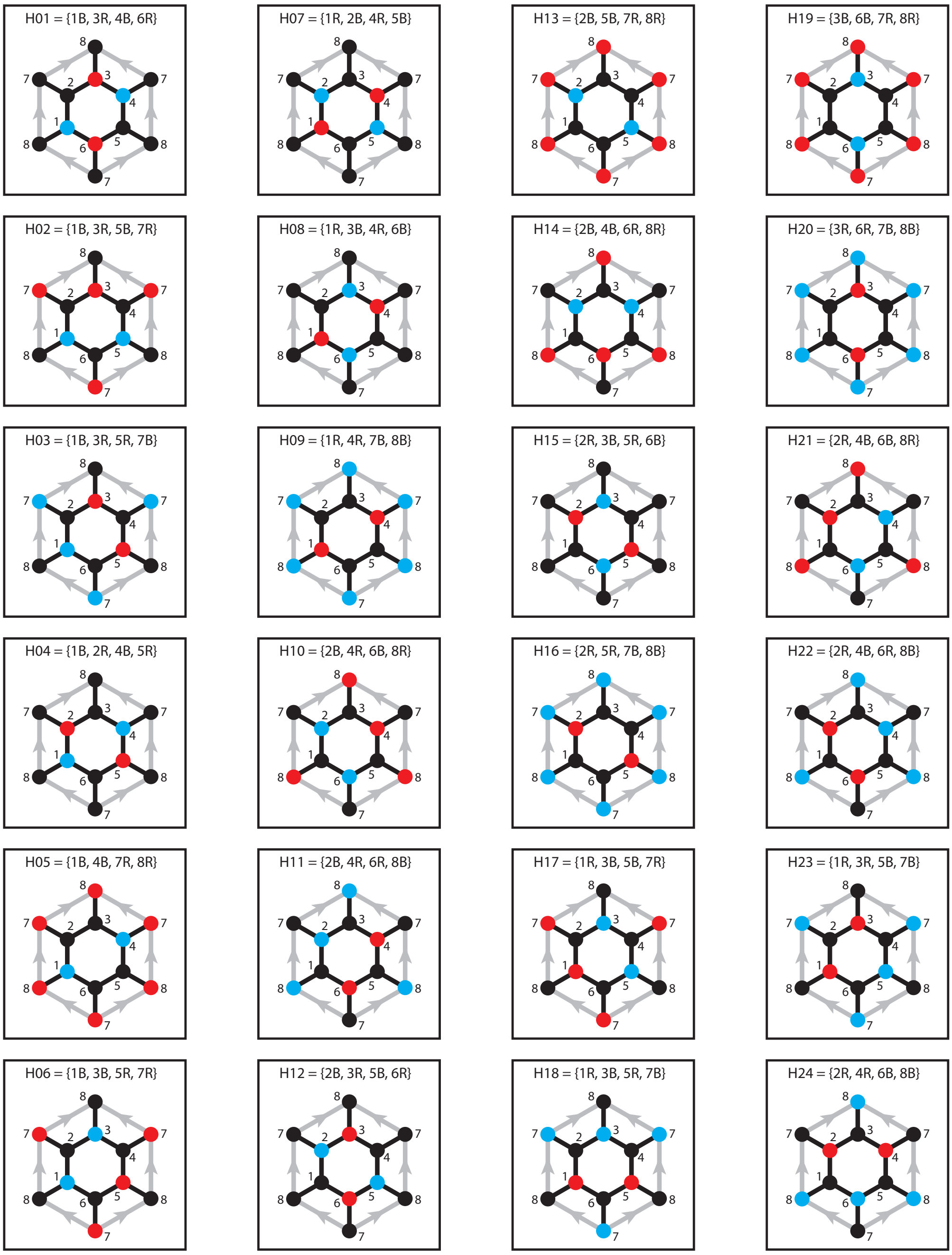

Figure 6: List of configurations of cardinality 4 corresponding to the hexagonal unit cell.

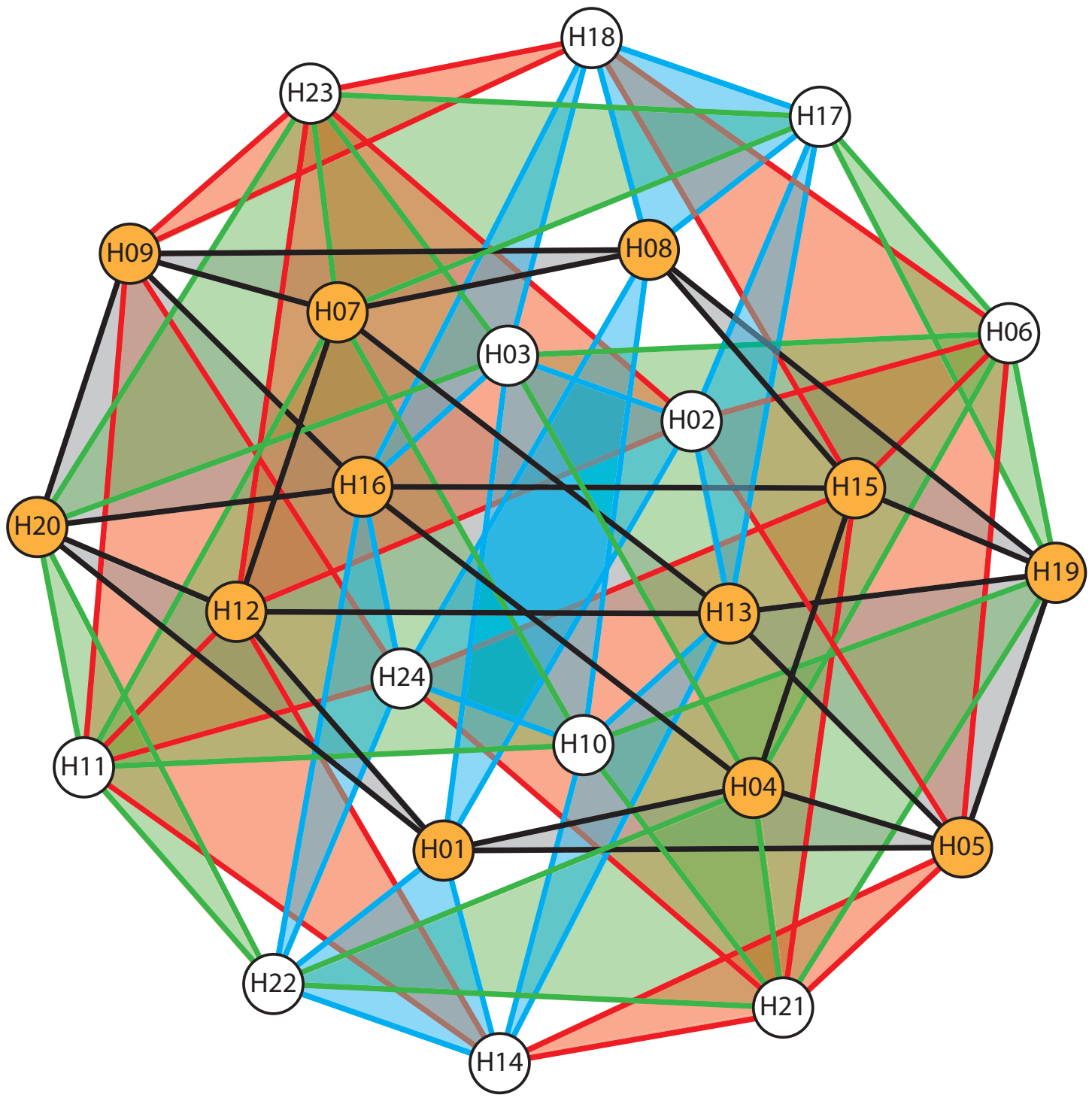

Figure 7: Correlation hypergraph corresponding to the hexagonal unit cell. Blue, red and green triangles are hyperedges associated with alveoli in directions 10, 01 and 11 respectively. Black triangles correspond to finite alveoli.
